# Quantifying requirements for mitochondrial apoptosis in CAR T killing of cancer cells

**DOI:** 10.1038/s41419-023-05727-x

**Published:** 2023-04-13

**Authors:** Alexandra L. Pourzia, Michael L. Olson, Stefanie R. Bailey, Angela C. Boroughs, Aditi Aryal, Jeremy Ryan, Marcela V. Maus, Anthony Letai

**Affiliations:** 1 Harvard Medical School MD-PhD Program, Boston, MA USA; 2 Dana Farber Cancer Institute, Division of Hematologic Neoplasia, Boston, MA USA; 3 Cellular Immunotherapy Program, Massachusetts General Hospital Cancer Center, Boston, MA USA; 4 Harvard Medical School, Boston, MA USA; 5 Present Address: Stanford Internal Medicine Residency, Palo Alto, CA USA; 6 Present Address: ArsenalBio, South San Francisco, CA USA

**Keywords:** Cancer, Cell death

## Abstract

Chimeric antigen receptor (CAR) T cell therapy is an FDA-approved treatment for several hematologic malignancies, yet not all patients respond to this treatment. While some resistance mechanisms have been identified, cell death pathways in target cancer cells remain underexplored. Impairing mitochondrial apoptosis via knockout of Bak and Bax, forced Bcl-2 and Bcl-XL expression, or caspase inhibition protected several tumor models from CAR T killing. However, impairing mitochondrial apoptosis in two liquid tumor cell lines did not protect target cells from CAR T killing. We found that whether a cell was Type I or Type II in response to death ligands explained the divergence of these results, so that mitochondrial apoptosis was dispensable for CART killing of cells that were Type I but not Type II. This suggests that the apoptotic signaling induced by CAR T cells bears important similarities to that induced by drugs. Combinations of drug and CAR T therapies will therefore require tailoring to the specific cell death pathways activated by CAR T cells in different types of cancer cells.

## Introduction

Chimeric antigen receptor (CAR) T therapy has revolutionized treatment options for patients with B cell malignancies. Its greatest successes have been in relapsed and refractory diffuse large B cell lymphoma (DLBCL) and B cell acute lymphoblastic leukemia (B-ALL), where many patients have experienced durable remissions of their disease [1–3]. However, not all patients with hematological malignancies respond to CAR T therapy, and solid tumors have proven even more difficult to treat [3–6]. One of the best studied reasons why CAR T therapy can fail is due to loss of target antigen expression in cancer cells [3]. However, events following T-cell recognition of target cells have been relatively less studied as mechanisms of resistance.

Aberrations in one cell death pathway, apoptosis, are frequent in hematological malignancies, and are associated with reduced sensitivity to chemotherapeutics and targeted agents [7–11]. Apoptosis occurs via two main pathways: the intrinsic pathway, which requires the mitochondrion; and the extrinsic pathway, which is initiated by cell surface death receptors like TNFR and FAS, and may or may not require recruitment of the intrinsic pathway to kill a cell [12, 13]. Intrinsic apoptosis is regulated by members of the Bcl-2 family of proteins [14–16]. This protein family is composed of pro- and anti-apoptotic proteins, and the interactions between these two groups determine cell fate [14, 15].

The extrinsic apoptotic pathway is triggered by the binding of death ligands to death receptors on the cell surface, triggering the activation of caspase 8 [12, 13, 17]. Caspase 8 subsequently cleaves both downstream caspases and Bid, and the anti-apoptotic protein XIAP determines whether downstream caspase cleavage is sufficient to lead to cell death [18]. Cells with high levels of XIAP following death ligand exposure are known as Type II cells, and require activation of mitochondrial apoptosis via Bid in order to die in response to death ligands. This contrasts with Type I cells, which maintain low levels of XIAP and do not require the mitochondrion to undergo extrinsic apoptosis [17].

Cytotoxic T cells (CTLs) can activate numerous cell death pathways in target cells, including apoptosis. Two well studied mechanisms by which CTLs kill include the release of perforin and granzymes, as well as the expression of death ligands. Human granzyme B has a high affinity for the Bcl-2 family member Bid, through cleavage of which it can activate intrinsic apoptosis in target cells [19–23]. It can also cleave downstream caspases directly, activating apoptosis independently from the mitochondrion [19–21]. Death ligands activate extrinsic apoptosis in target cells, which in some cells will also require activation of the mitochondrial apoptotic pathway in order to kill the target cell.

Previous work has implicated mitochondrial apoptosis in CTL killing of target cancer cells in models of immunotherapy. Ravi and colleagues demonstrated that cancer cells lacking Bax or expressing Bcl-XL were protected from in vitro and in vivo elimination by murine splenocytes [24]. In contrast, Pardo and colleagues demonstrated that target cancer cells lacking Bid, downstream caspases, and Bak/Bax are still capable of being killed by murine CTLs [25, 26]. This discrepancy remains unresolved, and the role of intrinsic apoptosis in target cell elimination by CTLs remains unclear.

Focusing in on CAR T cells as a specific subset of CTLs, conflicting data remain as to the mechanism by which they kill target cells. Singh and colleagues recently reported an in vitro CRISPR/Cas9 screen in the Nalm6 B cell leukemia cells to identify proteins whose loss conferred a survival benefit to target cells after exposure to CD19-directed CAR T cells [27]. They demonstrated that loss of death receptor signaling components conferred resistance to CAR T cells in vitro. This points to a potential mechanism of resistance to CAR T cells that remains underexplored—alteration of cell death pathways in target cancer cells. However, requirements for mitochondrial apoptosis, which in some cell types is required downstream of extrinsic apoptosis for death receptor killing of target cells, were not identified. In addition, there are multiple redundancies in the mitochondrial apoptotic pathway which may have obscured an effect in a CRISPR screen. This was also difficult to reconcile with previously published work demonstrating increased cytotoxicity when combining the BH3 mimetic ABT-737 with CAR T co-culture in vitro in a panel of pre-B-ALL cell lines [28, 29]. Thus, the role of mitochondrial apoptosis in CAR T killing remains unclear.

In order to determine if target cell mitochondrial apoptosis could alter CAR T cell killing efficacy, we generated several in vitro model systems with intact and deficient mitochondrial apoptosis. We then exposed target cells to CD19 CAR T cells and compared cytotoxicity in each. By estimating the “E:T50”, or effector to target cell ratio required to achieve 50% maximum killing of target cells, we were able to quantify the impact of mitochondrial apoptosis on target cancer cell death. This is the first test of an absolute requirement for intrinsic apoptosis in CAR T killing of cancer cells. Our results prompt a generalizable model that is not only consistent with our results, but also brings clarity to the apparent discrepancies in prior work on this topic.

## Results

**Fig. 1:**
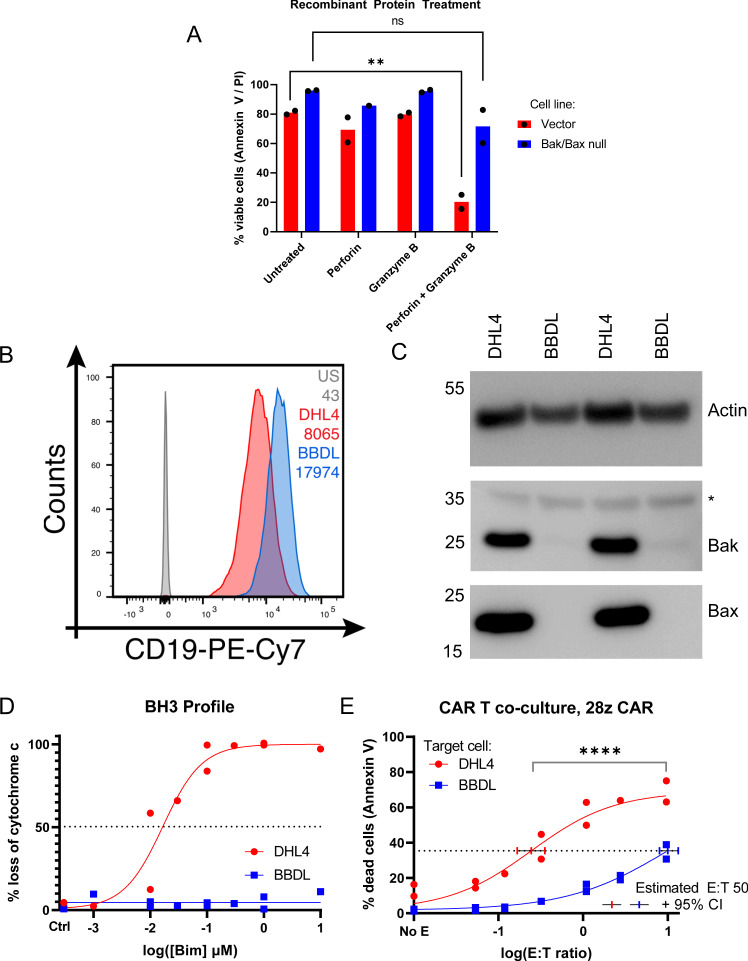
Loss of Bak and Bax is associated with resistance to granzyme B cytotoxicity and CAR T killing. **A** Annexin V / PI staining of vector and Bak/Bax null pre-B cell lines following exposure to perforin and/or granzyme B. *N* = 2, each point is a biological replicate. Significance corresponds to unpaired *t* test, **p = 0.003. **B** Quantification of CD19 staining intensity via flow cytometry in two lymphoma cell lines, DHL4 and BBDL. **C** Immunoblotting for Bak and Bax, with actin included as a loading control. **D** BH3 profiling of DHL4 and BBDL cell lines, *N* = 2, each point is a biological replicate. **E** Annexin V viability assay of DHL4 and BBDL cells 18 h after co-culture with CD19 CAR T cells. *N* = 2, each point is a biological replicate. Significance corresponds to unpaired *t* test, ****p < 0.0001.

In order to assess whether the mitochondrial pathway of apoptosis in target cancer cells could impact the killing efficacy of CAR T cells, we first generated in vitro model systems that approximated T cell killing of target cells more generally. We utilized an isogenic pre-B BCR-ABL ALL murine model with intact (Vector) and deficient (Bak/Bax null) mitochondrial apoptosis [30]. These target cells were exposed to recombinant human perforin and granzyme B for one hour in vitro, and viability was assessed at 4 and 20 hours after treatment (Fig. 1A). We observed that Bak/Bax deficient target cells were significantly more resistant to recombinant granzyme B-induced death than their Bak/Bax intact counterparts, consistent with a requirement for mitochondrial apoptosis in human granzyme-B induced cytotoxicity.

Having established that Bak/Bax deficiency protects from one component of CTL cytotoxicity, we next asked whether Bak/Bax deficiency was associated with protection from CAR T killing in a human lymphoma in vitro system. We utilized two lymphoma cell lines, SU-DHL-4 and BBDL, which express similar levels of CD19 (Fig. 1B). The BBDL cell line harbors a loss of Bak and Bax expression, as demonstrated via immunoblotting (Fig. 1C). We first confirmed that the BBDL cell line is resistant to mitochondrial apoptosis. To do this, we performed BH3 profiling, which quantifies how close a cell is to undergoing mitochondrial outer membrane permeabilization (MOMP) [31]. We observed that while the SU-DHL-4 cell line undergoes MOMP in response to 0.1 μM Bim peptide, the BBDL cell line was refractory to MOMP at all peptide concentrations tested (Fig. 1D). This confirms that BBDL cells are resistant to death occurring via mitochondrial apoptosis.

To test whether defects in mitochondrial apoptosis could protect cancer cells from CAR T killing, we generated an in vitro co-culture system. CAR T cells used in these experiments were generated from PBMCs from normal human donors, and contained one of three intracellular domains—28z, BBz, or delta zeta negative control (see methods), as described previously by Boroughs et al. [32]. Transduction efficiency varied between 30 and 90% amongst donors and CAR constructs (Fig. S1A). CAR+ cells were not sorted following transduction, and effector:target ratios were calculated using the number of CAR+ cells. Their specificity for killing CD19+ cells is described in Boroughs et al. [32]. For 11 donors where co-cultures were performed with both costimulatory domains, 6 donors exhibited roughly equal cytotoxicity with both CARs (Fig S1B). In the 5 remaining donors, one of the two CARs did not achieve specific killing above the level of the delta zeta negative control. These CAR T cells failed to achieve a threshold for specific cytotoxicity, and were thus excluded from experiments in order to focus on specific killing.

Target cells were labeled with CFDA-SE and incubated with CD19 CAR T cells at various effector to target cell (E:T) ratios for 18 h, and viability was assessed via annexin V / Hoechst staining and flow cytometry. In order to quantify relative cytotoxicity, we compared the E:T50 between each target cell line, which we define as the absolute E:T ratio at which 50% of maximum target cell death was achieved, estimated via non-linear regression.

We observed that SU-DHL-4 cells were rapidly eliminated by CD19 CAR T cells, with an E:T50 of 0.244 at 18 h. In contrast, BBDL cells were resistant to CAR T cytotoxicity, with an E:T50 of 9.97 (Fig. 1E). Thus in order to achieve the same level of target cell killing, Bak/Bax deficient cells required approximately 40 times as many CAR T cells compared to Bak/Bax intact counterparts. This data is consistent with defects in target cell mitochondrial apoptosis conferring protection from CAR T cells. However, the BBDL and SU-DHL-4 lines are not isogenic, so other factors could also contribute to BBDL resistance.

We therefore next tested the requirement for mitochondrial apoptosis in CAR T killing in an isogenic tumor model system. Our laboratory used HeLa cells to generate isogenic Bak/Bax double knockout cells (HeLa-DKO). We then transduced these cells to generate paired HeLa and HeLa-DKO CD19 expressing cell lines, known as HeLa-19 and HeLa-DKO-19, respectively. These cell lines expressed similar levels of CD19 and retained their Bak/Bax status, as ascertained by immunoblotting (Fig. S2A, S2B).

**Fig. 2:**
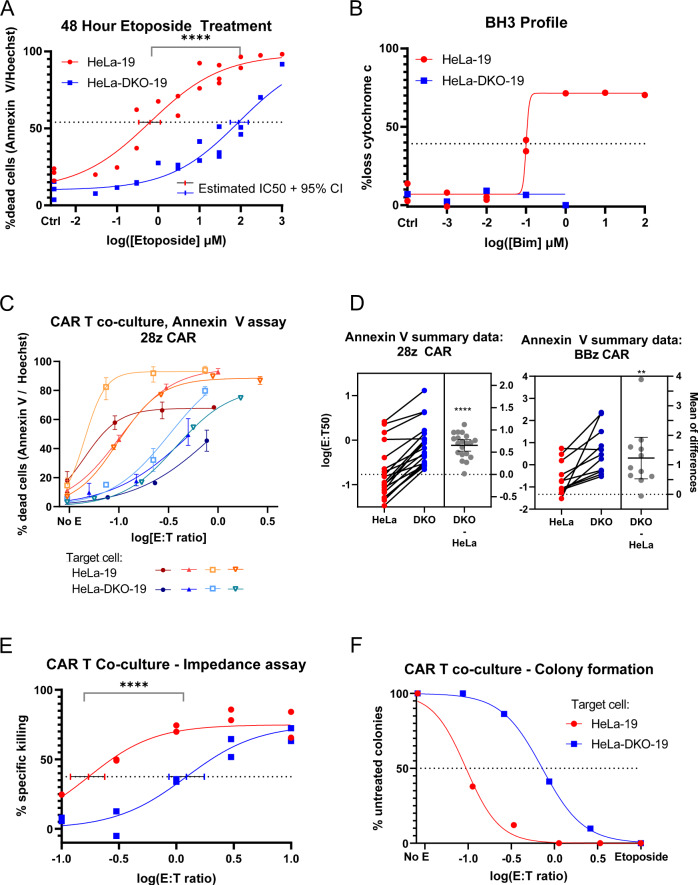
Loss of Bak and Bax confers resistance to CAR T killing of HeLa target cells. **A** Annexin V / Hoechst viability staining of HeLa-19 and HeLa-DKO-19 cells following 48 hour exposure to etoposide, *N* = 3. **B** BH3 profiling assay comparing mitochondrial response to the Bim peptide in HeLa-19 (*N* = 2) and HeLa-DKO-19 (*N* = 1) cell lines. Figure **C**–**F** depicts target cell viability following exposure to CD19 CAR T cells assessed via annexin V/Hoechst staining at 20 h (**C**, **D**), an impedance assay (**E**, *N* = 2), and a colony formation assay (**F**, *N* = 1). **C** presents data from 4 biological replicates using a single donor’s CAR T cells. Curves were fitted with non-linear regression, error bars are technical replicates. All biological replicates are shown in Fig. S2. The left panel of **D** presents the E:T 50 value for every paired co-culture experiment performed (*N* = 20 for 28z, *N* = 12 for BBz, 9 donors total). The right panel presents the mean difference in E:T50 (DKO-HeLA, middle bar) with the 95% confidence interval. For all graphs except panel **C**, each point is a biological replicate. Significance corresponds to either an unpaired (**A**, **E**) or paired (**D**) *t* test, ****p < 0.0001, **p = 0.0015.

In order to functionally demonstrate that HeLa-19 and HeLa-DKO-19 cells differ in their propensity towards undergoing mitochondrial apoptosis, we first assessed sensitivity to etoposide, a classical inducer of apoptosis (Fig. 2A). At low doses of etoposide (<1 µM), HeLa-19 cells were more sensitive to etoposide than their Bak/Bax null counterparts. However at higher doses, HeLa-DKO cells were killed as well. The estimated IC50 for each cell line was 0.632 µM for HeLa-19 and 88.7 µM for HeLa-DKO-19 cells. We concluded that HeLa-19 cells are more sensitive to death induced by etoposide when compared to HeLa-DKO cells, but the double knockout cells did not gain absolute protection. At higher doses, etoposide may activate non-apoptotic cell death in Bak/Bax deficient cells. We also used another functional measure of apoptotic susceptibility, the BH3 profiling assay, to confirm that HeLa-DKO-19 cells are resistant to mitochondrial apoptosis (Fig. 2B). As demonstrated previously in the BBDL cell line, HeLa-DKO-19 mitochondria were more resistant to the Bim peptide compared to their HeLa-19 counterparts.

Having generated two cell lines, HeLa-19 and HeLa-DKO-19, that differ in their susceptibility to mitochondrial apoptosis, we wanted to ask if they differed in their response to CD19 CAR T cells. To answer this question, we first quantified target cell viability 20 h after exposure to CAR T cells via annexin V/Hoechst staining. We again used non-linear regression to fit curves and estimate E:T 50 s for each paired experiment. To demonstrate this methodology, we depict all results from a single donor in Fig. 2C/S2C, with an example curve fit shown in Fig. S2D. From these two donors, a trend began to emerge—HeLa-DKO-19 target cells generally required more CAR T cells to achieve similar killing compared to their HeLa-19 counterparts.

We performed co-culture annexin V viability assays with CAR T cells generated from a total of 9 separate donors using both 28z and BBz co-stimulatory domains. These results are summarized in Fig. 2D, which presents estimated E:T50 values from every paired experiment. From this summary, we conclude that both 28z and BBz co-stimulatory domains recapitulated the same pattern, with HeLa-19 cells exhibiting greater susceptibility to CD19 CAR T cells than HeLa-DKO-19 cells across all donors tested. On average, HeLa-DKO-19 cells required 10.7 times more CAR T cells compared to HeLa-19 counterparts to achieve a similar level of killing. This difference in susceptibility is thirteen times less than their difference in susceptibility to etoposide-induced death (140 fold), as demonstrated in Fig. 2A.

To confirm these findings, we also assessed viability following co-culture via two additional methods: an impedance-based assay (Fig. 2E and S2E, E:T50s 0.172/1.22) and a colony formation assay (Fig. 2F, E:T50s 0.0947/0.722). Both of these methods corroborated our findings above, with HeLa-DKO-19 cells requiring about 7 times as many CAR T cells to achieve similar killing to HeLa-19 counterparts.

**Fig. 3:**
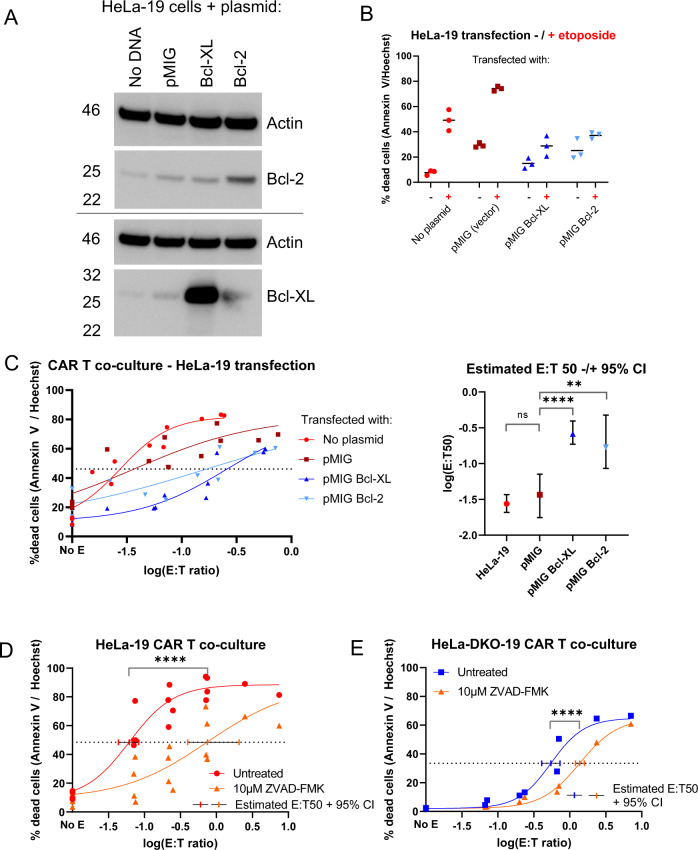
Forced expression of Bcl-2 and Bcl-XL confers resistance to CAR T killing of HeLa target cells. **A** Immunoblotting for Bcl-2, Bcl-XL, or actin (loading control) following transfection of HeLa-19 cells. **B** Annexin V / Hoechst viability staining at 20 h following etoposide treatment of each transfected population (*N* = 3). **C** Left panel: Annexin V / Hoechst viability staining of each cell population following 20 h of CAR T co-culture (*N* = 3). Right: estimated E:T 50 values −/+ 95% confidence interval for each curve, with significance calculated from unpaired t test, Bonferroni corrected for three comparisons. **D**, **E** Annexin V / Hoechst staining of HeLa-19 (**D**, *N* = 4) and HeLa-DKO-19 (**E**, *N* = 2) target cells following CAR T co-culture −/+ caspase inhibition with Z-VAD-FMK. Each point is a biological replicate, significance calculated from unpaired *t* test, ****p < 0.0001, **p = 0.0013.

We next perturbed other mediators of apoptosis to test if we could recapitulate effects of loss of the key apoptotic mediators Bak and Bax in HeLa-19 cell lines. First, we generated HeLa-19 cells that transiently expressed a control vector (pMIG) or the anti-apoptotic proteins Bcl-2 or Bcl-XL (Fig. 3A). We confirmed that Bcl-2 and Bcl-XL expression protected target cells from death induced by etoposide (Fig. 3B). We then performed co-culture experiments, incubating each HeLa-19 variant with CD19 CAR T cells (Fig. 3C). Both Bcl-XL expression (E:T50 0.259) and Bcl-2 expression (E:T50 0.169) protected target cells from CAR T killing, compared to the pMIG vector control variant (E:T50 0.0368, E:T50 fold change 4.59–7.05).

Caspases are important mediators of apoptotic killing. We next asked whether caspase inhibition with Z-VAD-FMK, a pan-caspase inhibitor, could protect target cells from CAR T killing (Fig. 3D, E, Fig. S3). Caspase inhibition protected both HeLa-19 (Fig. 3D, E:T50 fold change 12.2) and HeLa-DKO-19 (Fig. 3E, fold change 2.51) target cells from CAR T cytotoxicity, suggesting that CAR-T killing in HeLa-19 and in whatever non-intrinsic apoptotic process proceeds in HeLa-DKO-19 cells both involve the activity of downstream caspases, albeit to a lesser extent in the Bak/Bax null condition.

**Fig. 4:**
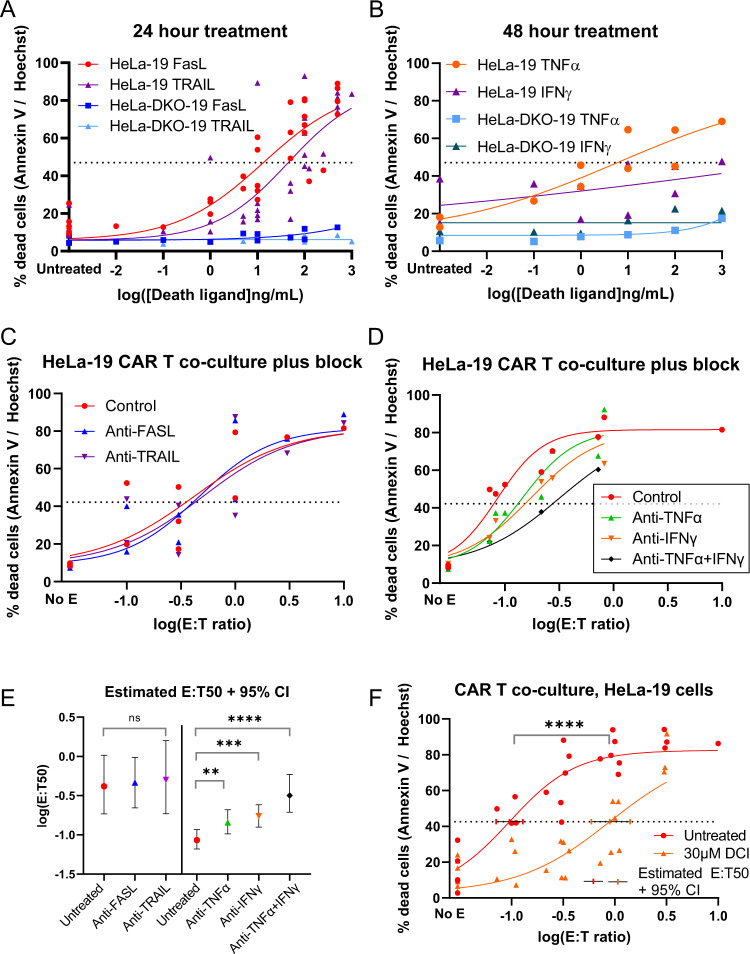
CAR T killing of HeLa target cells is mediated by granzymes, TNFα, and IFNγ. **A**, **B** Annexin V/Hoechst viability staining of HeLa-19 and HeLa-DKO-19 cells following 24 h (**A**, HeLa *N* = 5, DKO *N* = 2) or 48 h (**B**, HeLa *N* = 2, DKO *N* = 1) of treatment with the indicated death ligand or cytokine. **C**, **D** Results of Fas, TRAIL, and TNFα blockade in CAR T co-culture experiments. For **C** *N* = 3, for **D** Control *N* = 3, Anti-TNFα *N* = 2, Anti-IFNγ *N* = 2, Anti-TNFα + IFNγ *N* = 1. The estimated E:T50 values and 95% confidence intervals are presented in **E**. In **F**, target cells were co-cultured with CD19 CAR T cells pre-treated with 3,4 dichloroisocoumarin (DCI), *N* = 6. Viability was assessed by Annexin V / Hoechst staining at 24 h. Each point is a biological replicate, significance corresponds to unpaired *t* test, **p = 0.0066, ***p = 0.0008, ****p < 0.0001.

Both perforin/granzyme and death ligand mechanisms can potentially activate mitochondrial apoptosis. In order to better understand why HeLa-19 cells were protected by defects in mitochondrial apoptosis, we tested requirements for both of these mechanisms. We first assessed whether HeLa-19 and HeLa-DKO-19 cells were capable of being killed by recombinant death ligands and IFNγ (Fig. 4A, B, Fig. S4A–B). IFNγ, a secreted cytokine, was included in this panel based on data from a murine PD-1 blockade model demonstrating that it is capable of killing target cancer cells via ferroptosis [33]. HeLa-19 cells were eliminated by FasL and TRAIL within 24 h, while TNFα and IFNγ cytotoxicity occurred only at 48 h. In contrast, HeLa-DKO-19 cells were more resistant to recombinant death ligand and IFNγ treatment at all doses tested (Fig. S4A–B). For example, estimated IC50s for FasL at 48 hours were 0.224 for HeLa-19 cells, compared to 220 for HeLa-DKO-19 cells (fold change 983, *p* < 0.0001). This confirmed that HeLa-19 cells are Type II cells, which require the activation of mitochondrial apoptosis in order to be eliminated by death ligands.

In order to test whether death ligands and IFNγ are components of CAR T killing of HeLa target cells, we performed co-culture experiments in the presence of blocking antibodies against Fas, TRAIL, TNFα, and IFNγ (Fig. 4C–E; Fig. S4C). We did not observe a protective effect from blocking Fas or TRAIL in any of three independent experiments tested. Blockade of TNFα and IFNγ demonstrated a modest but statistically significant protective effect, with E:T50 fold changes of 1.69 for TNFα, 2.01 for IFNγ, and 3.71 for both in combination (Fig. 4D, E).

We hypothesized that an additional mediator of CAR T-induced cytotoxicity could be granzymes, which require cell-cell contact in order to be delivered to the target cell. To test a requirement for granzyme-induced cytotoxicity in our model, we pre-treated CAR T cells with the serine protease inhibitor 3,4-dichloroisocoumarin (DCI) prior to co-culture experiments. We first confirmed that DCI reduced granzyme B cleavage of substrates using an ex vivo fluorometric assay (Supplemental Fig. 5A). Next, we pre-treated CAR T cells with DCI prior to co-culture with HeLa-19 and HeLa-DKO-19 target cells. Both HeLa-19 (fold change 9.35) and HeLa-DKO-19 (fold change 4.37) cells were protected from CAR T killing in this setting (Fig. 4F, Fig. S5B–C). Taken together with our earlier finding that caspase inhibition protects HeLa-19 and HeLa-DKO-19 target cells from CAR T killing, this suggests that granzymes, particularly granzyme B, are another component of CAR T killing in our model system. Based on the magnitude of this protective effect compared to that of TNFα or IFNγ blockade, it appears that granzyme-mediated killing accounts for the majority of the cytotoxicity we observe in our HeLa-19 model system.

**Fig. 5:**
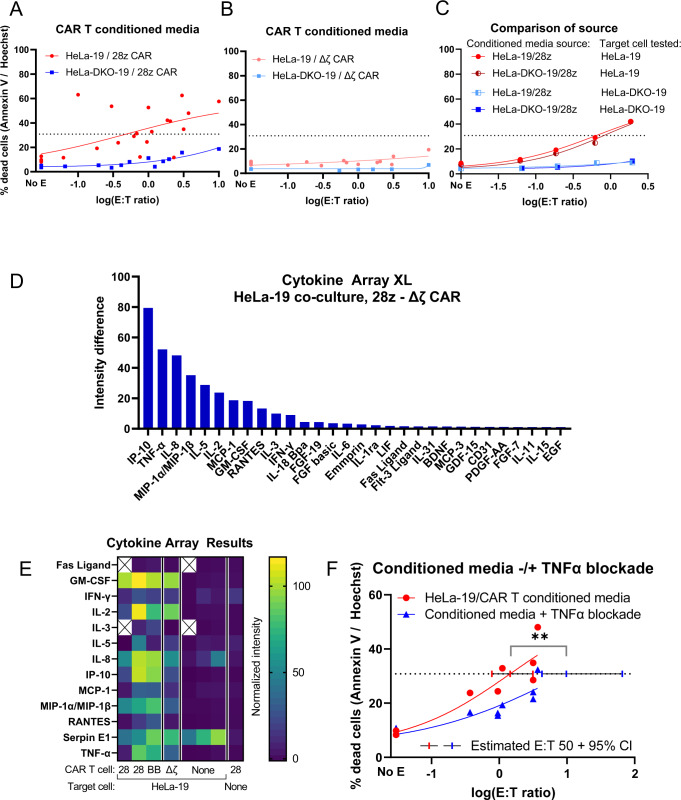
CAR T killing of HeLa target cells is mediated by soluble factors including TNFα. **A**, **B** Conditioned media was collected as described in the text and incubated with HeLa-19 or HeLa-DKO-19 target cells for 24 h. For **A**, HeLa *N* = 6, DKO *N* = 3; in **B** HeLa *N* = 4, DKO *N* = 1. In **C**, conditioned media from two paired co-cultures (HeLa-19 or HeLa-DKO-19 with 28z CAR T cells) was incubated with HeLa-19 or HeLa-DKO-19 target cells for 24 h. *N* = 1. Figures **D** and **E** present Cytokine Array data from conditioned media with intensity normalized to the included positive and negative controls. Figure **D** highlights cytokines that were unique to HeLa-19 co-culture with a specific CAR by subtracting the cytokines present in HeLa-19/Δζ co-culture (*N* = 1). Figure **E** presents results from all three cytokine arrays performed, highlighting the top cytokines noted in Figure **D**. **F** Annexin V / Hoechst viability assay performed 24 h after HeLa-19 incubation with conditioned media and the indicated blocking antibodies, *N* = 3. Each point represents a biological replicate, significance corresponds to unpaired *t* test, **p = 0.0017.

In order to better assess whether CAR T killing was mediated by soluble factors we did not test above, we generated CAR T conditioned media from co-culture with HeLa-19 cells. We first tested whether this conditioned media was cytotoxic to either target cell type. HeLa-19 cells exhibited a cytotoxic response to conditioned media generated from HeLa-19/CAR T co-culture, whereas HeLa-DKO-19 cells were resistant to conditioned media from HeLa-DKO-19/CAR T co-culture (Fig. 5A, fold change E:T50 > 20.8). Conditioned media from co-culturing HeLa-19 and DKO-19 target cells with a negative control CAR led to minimal cytotoxicity in comparison, although HeLa-19 target cells still exhibited more cell death than their DKO-19 counterparts (Fig. 5B). However, when HeLa-19 cells were exposed to conditioned media from HeLa-DKO-19/CAR T co-culture, they were also eliminated (Fig. 5C). This suggests that the difference in target cell response to conditioned media can be attributed to differences in the target cell (Bak/Bax deficient versus wildtype) sensitivity to soluble factors, rather than in differences in the conditioned media generated by these two co-culture conditions. Once again, removing mitochondrial apoptosis protected HeLa-19 target cells from a form of CAR T cytotoxicity.

We then performed cytokine arrays to identify potentially cytotoxic factors present in CAR T conditioned media. The most abundant cytokines selectively present in CAR T co-culture with 28z CAR T cells, as opposed to delta zeta negative control CAR T cells, included IP-10, TNFα, IL-8, MIP1α/1β, IL-5, IL-2, MCP-1, and GM-CSF (Fig. 5D). The ranked list of all cytokines tested can be found in Supplemental Figs. S6–S8 and Supplemental Tables 2–4. Of potentially cytotoxic factors assayed by our cytokine arrays, both TNFα and IFNγ were detected in all three cytokine arrays performed (Fig. 5E). FasL was not detected in the two experiments where it was assessed, consistent with our previous data that Fas signaling blockade did not protect HeLa-19 cells from CAR T killing.

To test whether death ligands could be responsible for HeLa-19 death in response to conditioned media, we exposed HeLa-19 target cells to conditioned media in the presence of blocking antibodies against TNFα (Fig. 5F, Fig. S8B). TNFα blockade demonstrated a statistically significant protective effect, with an E:T50 fold change of 6.76. In conclusion, TNFα blockade protects HeLa-19 target cells from CAR T killing via conditioned media, but this protection is less than the protection we observed in the Bak/Bax null condition (Fig. 5A, fold change >20.8). This suggests that CAR T conditioned media cytotoxicity proceeds via additional mechanisms. Additionally, the magnitude of protective effect of antibody blockade was weaker in co-culture experiments (Fig. 4E) compared to conditioned media, confirming that other mechanisms of CAR T killing are active in direct co-culture experiments (such as granzymes, as demonstrated in Fig. 4).

**Fig. 6:**
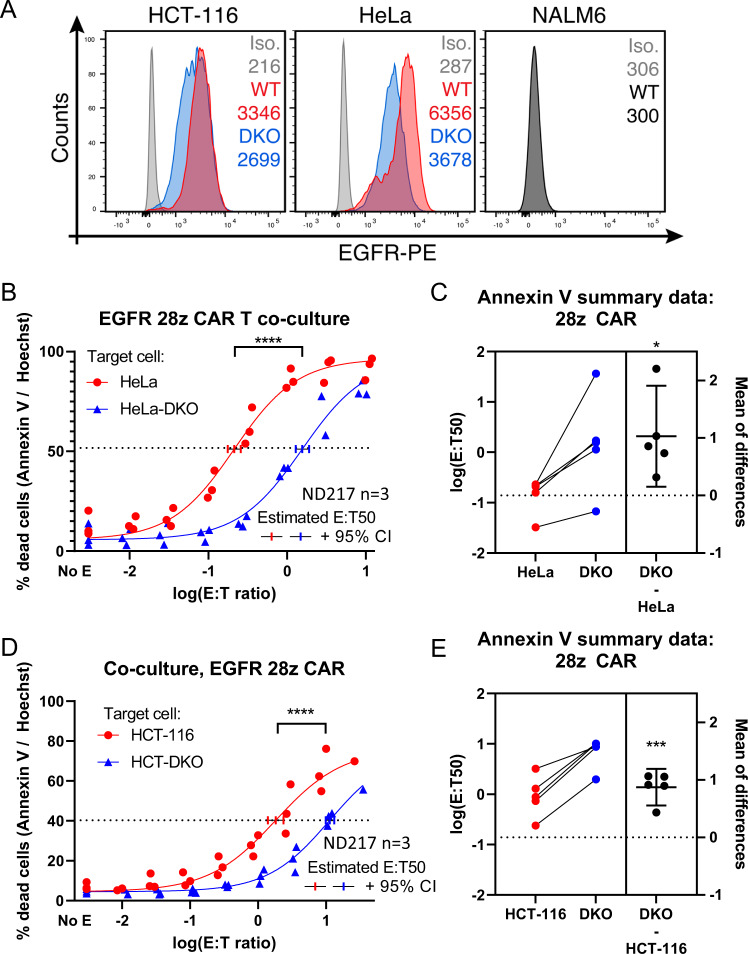
Loss of Bak and Bax confers resistance to CAR T killing in solid tumor target cells. **A** EGFR expression quantified by flow cytometry for paired WT and DKO HCT-116 and HeLa cell lines. Nalm6 included as negative control, Iso = isotype control. Panels **B**–**E** depict target cell viability following exposure to EGFR CAR T cells assessed via annexin V/Hoechst staining at 20 h. **B**, **D** Pooled data from biological replicates using a single donor’s CAR T cells, with a curve fit using non-linear regression. In **C** and **E**, curves were fit for each biological replicate performed, and E:T 50 values are presented for each wildtype/DKO pair (total of 3 donors and 5 replicates). Each point is a biological replicate, significance corresponds to either an unpaired (**B**, **D**) or paired (**C**, **E**) *t* test. *p = 0.0156, ***p = 0.0008, ****p < 0.0001.

Having demonstrated that mitochondrial apoptosis impacts CAR T killing in one isogenic model, we wanted to determine if the same would hold true in additional models of CAR T therapy. Given that our HeLa-19 model pairs a solid tumor with a CAR targeting a protein expressed on hematopoietic cells, we asked whether the same results would hold true in a solid tumor model with a CAR T directed toward an innate antigen. We utilized two solid tumor cell lines, wildtype HeLa cells and HCT-116 cells, for which we had isogenic paired cell lines with Bak/Bax deficiency (Fig. S9A). Both of these tumor models express EGFR (Fig. S9B). We demonstrated that wildtype HeLa and HCT-116 target cells could be killed specifically by EGFR-directed CAR T cells (Fig. S9C–D, EGFR CARs further characterized in Boroughs et al. [32]). We then co-cultured wildtype HeLa and HeLa Bak/Bax null (DKO) target cells with EGFR-directed CAR T cells, and observed that loss of mitochondrial apoptosis conferred similar protection from CAR T killing as we observed in the HeLa-19 model (Fig. 6B). On average across three donors tested, the shift in the E:T50 between HeLa-DKO and wildtype HeLa cells was 10.7 fold (Fig. 6C). We next co-cultured wildtype and Bak/Bax null (DKO) HCT-116 target cells with EGFR-directed CAR T cells (Fig. 6D). Once again, our findings were similar to those in our HeLa-19 model: loss of Bak and Bax in HCT-116 cells conferred protection from CAR T killing, with an average shift in the E:T50 of 7.5 across three donors tested (Fig. 6E). Although the lower EGFR expression levels in the DKO variants likely contributes to a reduction in killing, the magnitude of expression difference is far less than the observed difference in E:T ratios between the two.

**Fig. 7:**
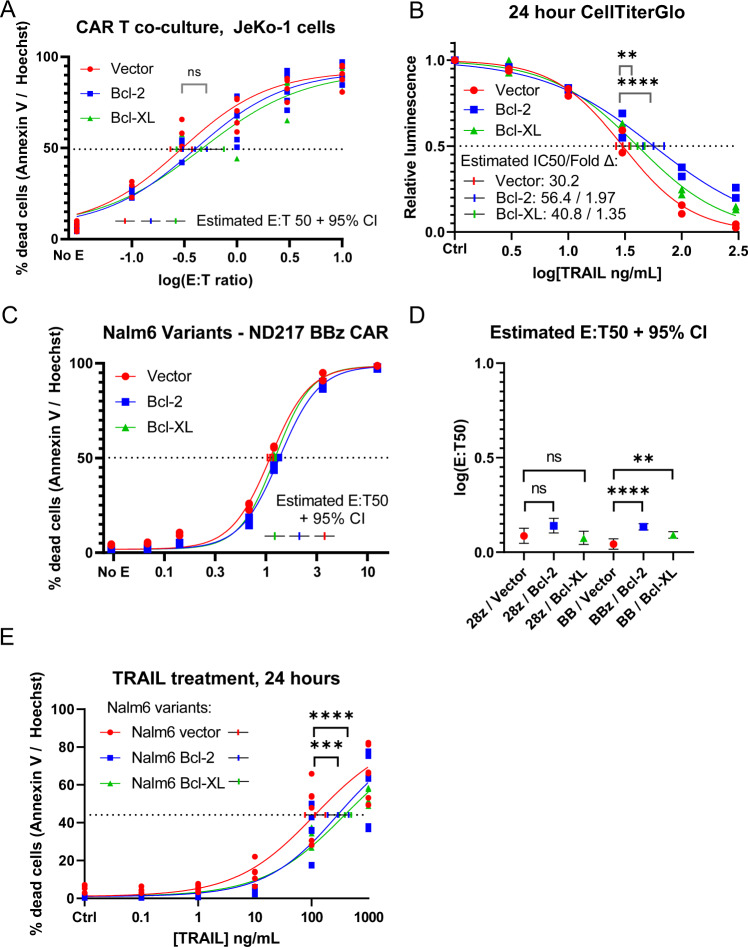
Loss of Bak and Bax confers minimal resistance to CAR T killing in lymphoma target cells. **A** Sensitivity of JeKo-1 variants to co-culture with CD19 CAR T cells (Vector *N* = 5, Bcl-2 *N* = 5, Bcl-XL *N* = 3) assessed by annexin V/Hoechst staining at 20 h. **B** Viability of JeKo-1 variants following 24 h of TRAIL treatment (*N* = 2). **C** Sensitivity of Nalm-6 variants to co-culture with CD19 BBz CAR T cells (*N* = 3, single donor) assessed by annexin V / Hoechst staining at 20 h. **D** Summary statistics for the biological replicates shown in C as well as for co-culture with 28z CD19 CAR T cells. **E** Viability of Nalm-6 variants following 24 hours of TRAIL treatment (*N* = 2). Each point is a biological replicate, significance corresponds to an unpaired (**A**, **B**, **D**, **E**) *t* test. **p = 0.0013 in **B**, =0.0017 in **D**, ***p = 0.009, ****p < 0.0001.

Given that the best-studied clinical use of CAR T cells is in hematological malignancies, we asked if an isogenic model expressing endogenous CD19 would yield similar findings to those in our solid tumor models. We utilized the JeKo-1 cell line, a CD19-expressing mantle cell lymphoma model (Fig. S10A). To block mitochondrial apoptosis, we generated JeKo-1 variants expressing Bcl-2, Bcl-XL, or a vector control via lentiviral transduction (Fig. S10B). The variants expressing Bcl-2 and Bcl-XL, but not the vector control cells, were protected from treatment with etoposide, an inducer of apoptosis (Fig. S10C, fold change in IC50 > 79, also S10D–E). To determine if Bcl-2 and Bcl-XL expression could protect JeKo-1 cells from CD19 CAR T killing, we developed a co-culture protocol similar to our methodology for HeLa-19 cells. In contrast to our findings above, JeKo-1 cells expressing Bcl-2 or Bcl-XL were not significantly protected from killing by CD19 CAR T cells compared to vector-expressing control cells (Fig. 7A, fold change in E:T50 1.36–1.55).

One possible explanation for the difference in protection conferred by inhibiting mitochondrial apoptosis between JeKo-1 and HeLa-19 cells could be their response to death ligands. HeLa cells are Type II cells, which require mitochondrial apoptosis in order to be killed by death ligands. To assess whether JeKo-1 cells were type I (death ligand killing independent of mitochondria) or type II in response to death ligands, we exposed the three JeKo-1 variants to recombinant FasL, TRAIL, and TNFα (Fig. 7B, Fig. S10F–J). The expression of Bcl-2 and Bcl-XL conferred a statistically significant but small survival benefit to JeKo-1 cells treated with TRAIL (fold change 1.35–1.87 with forced expression of Bcl-XL and Bcl-2, compared to >983 for Bak/Bax null HeLas). This demonstrates that these cells can die via extrinsic apoptosis independent of the mitochondrion, and thus are Type I cells. We assessed whether the addition of a TRAIL blocking antibody could protect JeKo-1 cells from CD19 CAR T cells in coculture (Fig. S10K). However, similar to our findings in HeLa-19 cells, we did not observe a significant protective effect following TRAIL blockade (E:T50 fold change 1.31–1.46), suggesting that TRAIL signaling was not required for CAR T killing of JeKo cells.

The possibility that CAR T killing in a Type I cell could proceed independently of the mitochondrion suggested a possible explanation for the findings of Singh et al. in the Nalm6 cell line [27]. We hypothesized that Nalm6 cells were also Type I in response to death ligands, given the identification of death receptor pathway member proteins identified as hits in both CAR T CRISPR screens. We also hypothesized that Nalm6 cells would be killed by CD19-directed CAR T cells independent of mitochondrial apoptosis, as we observed in JeKo-1 cells. To test these hypotheses, we utilized the same Bcl-2 and Bcl-XL lentiviral constructs used above to generate Nalm6 empty vector, Nalm6 Bcl-2, and Nalm6 Bcl-XL expressing variants (Fig. S11A). Similar to JeKo-1 cells, the Bcl-2 and Bcl-XL expressing Nalm6 variants were protected from treatment with etoposide (Fig. S11B, fold change > 380 for Bcl-2 and Bcl-XL expression). However, although Bcl-2 and Bcl-XL expression provided statistically significant protection from CAR T killing compared to empty vector control cells, the magnitude of this protection was only 1.1-1.3 fold (Fig. 7C, D, S11C). Additionally, we exposed Nalm6 cells to recombinant TRAIL, and observed that although Bcl-2 and Bcl-XL expression conferred statistically significant protection from TRAIL, the magnitude of this effect was small (Fig. 7E, fold change 2.5–3.4). Thus, Nalm6 cells are Type I cells, as we hypothesized previously.

## Discussion

While CAR T therapy has revolutionized treatment for relapsed and refractory B cell malignancies, more work needs to be done to improve upon these responses and to extend these results to additional tumor types. One strategy to achieve this is to better understand how CAR T cells kill different cancer cells, so that we can target relevant cell death pathways. Previous work implicated mitochondrial apoptosis in CAR T cell elimination of tumor cells by combining BH3 mimetics and CAR T cells in vitro [28]. However, this was difficult to reconcile with data from additional tumor models demonstrating that the intrinsic apoptotic pathway was dispensable for killing by CTLs [25, 26]. We sought to expand upon these data, and to clarify why intrinsic apoptosis seems to be involved in CAR T cell elimination of certain target cells, but not others.

In this work, we concluded that in certain contexts, CAR T cells do eliminate target cells via mitochondrial apoptosis. We demonstrated this by perturbing mitochondrial apoptosis in HeLa target cells in three different ways: by removing Bak and Bax; by forcing the expression of Bcl-2 and Bcl-XL; and by co-culturing CAR T and target cells in the presence of caspase inhibitors. All three methodologies demonstrated that impaired mitochondrial apoptosis reduced CAR T killing of target cells.

One important lesson is that which mechanisms of cell death dominated was a property of the target cell, rather than of the specific CAR T cells. In this study, we utilized CAR T cells generated from nine unique donors, as well as two second-generation CARs. By looking at this data collectively, we conclude that impairment of mitochondrial apoptosis was universally protective to HeLa-19 cells, in that removing Bak and Bax always conferred a survival benefit. The magnitude of the protection varied, but generally took the form of a five to ten-fold increase in the number of CAR T cells present in order to achieve similar killing compared to wild-type counterparts. For comparison, this was thirteen times less than to the protection conferred in Bak/Bax null cells following treatment with etoposide, a classical inducer of mitochondrial apoptosis. Thus, although Bak/Bax null cells are significantly protected from CAR T killing, CAR T cells appear to have the ability to activate non-apoptotic cell death in target cells to a greater extent than etoposide.

Thirdly, we demonstrated that the protective effect of impaired mitochondrial apoptosis is context dependent. Having demonstrated that impaired mitochondrial apoptosis protects HeLa-19 cells from CAR T killing, we observed similar results both in wildtype HeLa target cells and in a colorectal model using the HCT-116 cell line. However, when we perturbed mitochondrial apoptosis in two isogenic hematopoietic models using JeKo-1 and Nalm6 target cells, we observed minimal protection from CAR T killing. This builds upon work by Singh et al., which previously demonstrated that extrinsic apoptosis impacts CAR T killing in a Nalm-6 CRISPR screen, but did not yield similar results for intrinsic-pathway specific proteins [27]. Moreover, our work suggests that the answer to the apparent conundrum of whether or not mitochondrial apoptosis is activated by CAR T cells is that while Type II cells require the mitochondrial pathway of apoptosis for efficient CAR T killing, this pathway is dispensable for Type I cells. Both Nalm6 and JeKo-1 cells are type I, whereas Hela cells are type II. This context-dependency could also explain the different findings of Ravi and Jaime-Sanchez and colleagues, where impaired mitochondrial apoptosis hindered CTL-based killing in one model but not another [24, 25].

Finally, in the clinic one can broadly observe that CAR T cells have had greater efficacy in hematologic tumors than in solid tumors. In previous work, we have found generally that solid tumor cells are less primed for the mitochondrial pathway of apoptosis than hematologic malignancy cells [7]. While there are doubtless several properties leading to this contrast of clinical results in hematologic and solid tumors, we speculate that this general property of low apoptotic priming of solid tumors might offer another explanation for the broad reduction in CAR T cell efficacy observed in them so far.

In conclusion, we find that mitochondrial apoptosis is a significant component of CAR T cell killing of cancer cells, particularly in Type II cells. While targeting mitochondrial apoptosis could alter responses to CAR T therapy in some cancers, it is unlikely to be a universally effective strategy. CAR T killing of target cells is likely to involve the activation of multiple cell death pathways, as our data and recent work by Zhang and colleagues demonstrate [34]. Achieving optimal response to CAR T therapy across a variety of cancer cell types will likely require an in depth understanding of the pathways that are available and utilized in each.

## Materials and methods

### Experimental design

The objective of this project was to determine if target cancer cells with intact mitochondrial apoptosis were killed by CAR T cells more efficiently than counterparts with apoptotic deficiencies. A secondary objective was to determine the cytotoxic mechanisms (i.e., death ligands, granzymes, other) employed by CAR T cells in killing target cells.

### Cell lines and tissue culture

Suspension cell lines SU-DHL4, BBDL, and Nalm6 were cultured in RPMI medium supplemented with 10% fetal bovine serum (FBS), penicillin-streptomycin (pen-strep), and plasmocin. Vector control and Bak/Bax null murine pre-B ALL cell lines were a gift from Joseph Opferman (Memphis, TN USA). HeLa parental and Bak/Bax double knockout (DKO) cells were generated by Kris Sarosiek and Cameron Fraser in the Letai laboratory. HCT-116 parental and Bak/Bax double knockout (DKO) cells were a gift from Bert Vogelstein (Baltimore, MD USA). Parental and CD-19 expressing HeLa cells and HCT-116 cells were maintained in DMEM supplemented with 10% FBS, Pen-strep, and plasmocin. JeKo-1 mantle cell lymphoma cells were purchased from the ATCC (Manassas, VA USA) and transduced with a lentiviral vector to express luciferase-GFP. JeKo-1 cells were maintained in RPMI-20%FBS-PenStrep-Plasmocin. HEK 293T/17 cells were purchased from the ATCC and maintained in DMEM-10%FBS-PenStrep-Plasmocin. Cells kept in long-term culture were maintained in medium with anti-mycoplasma antibiotic (Plasmocin) and periodically tested negative for mycoplasma.

### CAR T cell generation

PBMCs from normal human donors were transduced with second generation CARs targeting CD19, similar to previously published protocols [35]. The CD19-directed CAR T cells used in this work contained one of two costimulatory domains, either 28z or 41BB, and a third CAR lacking the CD3 zeta chain (delta zeta) was used as a negative control. For CAR T co-culture experiments, CAR T cells were plated in RPMI supplemented with 10% FBS / pen-strep.

### Recombinant proteins

Recombinant human perforin and human granzyme B were purchased from Enzo Life Sciences (Farmingdale, NY USA). Additional recombinant proteins were purchased from: IFNγ— Peprotech (East Windsor, NJ USA), TNFα—Thermo Fisher Scientific (Waltham, MA USA), TRAIL—Biolegend (San Diego, CA USA), FasL—Enzo Life Sciences, and reconstituted according to the manufacturer’s instructions.

### In vitro perforin and granzyme treatment

Perforin was dissolved in 1%BSA-HEPES buffer, and granzyme B was dissolved in Ca-HEPES buffer. Perforin titrations were performed in a 96 well plate, with viability assessed via trypan blue staining. A sub-lytic concentration of perforin was chosen that led to less than 20% non-viable cells by trypan blue staining. Cells were incubated with perforin and / or granzyme B in Ca-/1%-BSA-HEPES buffer for 1 hour at 37 °C. Cells were then washed and plated in culture medium. Viability was assessed at 4 h and at 20 h via annexin V / PI staining.

### Annexin V / PI and Annexin V / Hoechst viability staining – suspension cell lines

For suspension cell lines, viability was assessed as follows. Annexin V with PI / Hoechst was diluted to 10x concentrate in 10x annexin V staining buffer (Ca-PBS). Cells were incubated with annexin V / PI / Hoechst for a minimum of 15 min. For Annexin V / PI staining, staining was quantified on a flow cytometer and dead cells were presented as 100%-% live quadrant via a quadrant gating system. For fixed Annexin V / Hoechst assays, cells were then fixed in glutaraldehyde/paraformaldehyde, the fixative was neutralized, and cells were analyzed via flow cytometry. Dead cells by annexin V / Hoechst were quantified as 100%-%live quadrant via a quadrant gating system.

### Co-culture assay with CD19 CAR T cells – DHL4 and BBDL

One day prior to co-culture, target cells were stained with CFDA-SE according to the manufacturer’s instructions. On the day of co-culture, CD19 CAR T cells were thawed and counted in RPMI-10%FBS. CAR T cells and target cells were plated at the indicated E:T ratios and incubated overnight at 37 °C. The next day, annexin V viability staining was performed as described above. Viability was quantified on a Fortessa-2 flow cytometer.

### CD19 staining

Cells were harvested and resuspended at 1E6/100 µL in FACS Buffer (FBS-HBSS) with blocking antibody and incubated for 10 min on ice. CD19 antibody was diluted 1:500 into cells and incubated for 30 min on ice. Cells were washed in FACS Buffer and CD19 staining intensity was quantified via flow cytometry.

### Immunoblotting

Lysates were prepared in RIPA buffer (Pierce, Waltham MA USA). For suspension cell lines, cells were pelleted and resuspended in RIPA buffer with protease and phosphatase inhibitor cocktails (commercially available) and incubated on ice for 5 min. For adherent cell lines, culture medium was aspirated and cells were rinsed once in cold PBS. RIPA buffer with protease and phosphatase inhibitor cocktails (commercially available) was added directly to the plate, and cells were incubated on ice for 5 min. Lysates were centrifuged at 10,000 RCF for 10 min, and the supernatant was removed to a fresh tube, discarding cellular debris and DNA in the pellet. Samples were prepared for immunoblotting via the addition of 4x Laemmli dye, DTT, and RIPA to yield a final protein concentration of 1 µg/µL. Samples were boiled for 5 min, then loaded on a Bis-Tris 4–12% gradient gel. Gel electrophoresis was performed at 100 mV for 1–2 h. The gel was then transferred to a PVDF membrane activated in methanol. Following transfer, membranes were blocked in 5% milk (in PBST). Membranes were washed 3 times for 10 minutes in PBST before incubation with primary antibodies overnight at 4 °C. The following day, membranes were washed in PBST and incubated with secondary antibodies in 5% milk for 1–2 h at room temperature. Membranes were washed in PBST and developed using SuperSignalWest ECL (Pierce). Digital imaging was performed on an LASImageQuant4000. Uncropped gels from all immunoblots are presented in Fig. S12. Primary antibodies were sourced from Cell Signaling Technologies (anti-actin 4970S, Bak 12105S, Bax 89477S, Bcl-2 15071S, Bcl-XL 2764S, EGFR 2232S, Vinculin 13901T). Secondary antibodies were sourced from Cell Signaling Technologies (Anti-rabbit 7074S, Anti-mouse 7076S).

### BH3 profiling – iBH3 methodology (flow cytometry)

Suspension cells were harvested in culture medium and washed in HBSS prior to resuspension in MEB2p25 buffer. Adherent cells were first trypsinized and washed in HBSS prior to resuspension in MEB2p25. Cells in MEB2p25 were added to 384 well peptide plates and incubated for 30 min at 27 °C. Cells were then fixed in formaldehyde, neutralized with N2 buffer, and incubated with cytochrome c antibody and Hoechst overnight at 4 °C. Cytochrome c retention or loss was quantified via flow cytometry.

### Generation of HeLa / HeLa-DKO cells expressing CD19

HeLa and HeLa-DKO cells were transduced with CD19-encoding lentivirus. Parental cells were incubated with viral supernatant and polybrene for 24 h. The media was then changed to fresh DMEM-10%FBS, and cells were rested and allowed to expand. Following expansion, cells were stained for CD19 as described above. CD19+ cells were sorted via FACS by the DFCI Flow Cytometry Core. Post-sorting, polyclonal populations of HeLa-19 and HeLa-DKO-19 cells were again rested in DMEM-10% FBS and allowed to expand.

### Annexin V / Hoechst viability staining – adherent cell lines

For adherent cell lines, viability was assessed as follows. The supernatant was first harvested and placed in a V-bottom 96 well plate. Cells were trypsinized and added to the supernatant in the same V-bottom plate. The plate was spun at 500rcf for 5 min to pellet cells. The supernatant was then aspirated, and cells were resuspended in the appropriate media (DMEM-10%FBS or RPMI-10%FBS for CAR T co-culture). Annexin V / Hoechst was added and cells were fixed in glutaraldehyde/paraformaldehyde as described previously. Fixed and neutralized cells were analyzed on a Fortessa 2 flow cytometer. Dead cells by annexin V / Hoechst were quantified as 100%-%live quadrant via a quadrant gating system.

### Co-culture assay with CD19 CAR T cells – flow cytometry viability assessment of adherent cells

One day prior to co-culture, target cells were stained with CFDA-SE according to the manufacturer’s instructions (ThermoFisher). HeLa-19 and HeLa-DKO-19 cells were seeded in 96 well plates at a density of 5000 cells per well in 100 µL of culture medium. On the day of co-culture, CD19 CAR T cells were thawed and counted in RPMI-10%FBS. CAR T cells were diluted to the corresponding E:T ratios and incubated with target cells overnight at 37 °C. The next day, annexin V viability staining was performed as described above. Viability was quantified on a Fortessa-2 flow cytometer.

### Curve fitting and IC50 / E:T 50 estimations

All viability data generated as described above were fit to a nonlinear curve using GraphPad Prism 9 software. Multiple biological replicates were plotted together, such that each point is a single biological replicate, which is the average of technical replicates. The x axis (E:T ratio or dose of compound) was transformed to a logarithmic scale, with the 0 T cell or untreated condition having an X value of a half log below the lowest E:T ratio or treatment plated. A nonlinear curve was fit using the following values: 4PL, x is log, top Y value set at the average maximum kill, bottom Y value set at the average minimum baseline cell death. The IC50 value of this curve provided the E:T 50, or the E:T ratio at which 50% of target cells were killed.

In order to present the variability in technical replicates, Fig. 2C and Supplemental Fig. S2C depict curves for individual experiments (*N* = 1), with error bars representing the range of technical replicates. In order to present the paired data in Figs. 2D, 6C, and 6E, curves were generated from single experiments via non-linear regression with the following settings: 4PL, x is log, top Y value < 100, bottom Y value = 0. IC50 values from curve fits with an R^2^ value greater than 80% were then presented in Fig. 2D.

### Co-culture assay with CD19 CAR T cells - Acea xCELLigence impedance readout of adherent cells

Target cells were seeded in proprietary 96 well plates at 5000 cells / well in 100 µL media and placed in the xCELLigence machine to measure impedance (time of seeding = 0 h). T cells were thawed, counted, diluted to various E:T ratios, and added to target cells 20 h later. Impedance measurements were performed every 15 min for a total of 72 h. An area under the curve was calculated for each well for hours ~22–72 (time of T cell addition to termination of experiment), and this number was normalized to negative control CAR T cell value as follows: |AUCsample - AUCdelta zeta | /(AUCdelta zeta) × 100. This experiment was performed two times, the graph presents normalized impedance for the two independent experiments.

### HeLa-19 transient transfection

Plasmids 3544 pMIG Bcl-2 and 3541 pMIG Bcl-XL were a gift from Stanley Korsmeyer (Addgene plasmid #8793, #8790; Watertown, MA USA). pMIG was a gift from William Hahn (Addgene plasmid # 9044). HeLa-19 cells were transfected using Lipofectamine 3000 reagent according to the manufacturer’s instructions. Briefly, cells were seeded at 4000 cells/well in a 96 well plate in antibiotic-free media (day 0). The following day, transient transfection was performed (day 1). Cells were incubated with plasmids overnight. On day two, the media was changed to fresh DMEM-10%FBS. On day 3, cells were treated with etoposide or CAR T cells as described previously. Finally, on day 4, cells were harvested and viability was assessed via annexin V / Hoechst staining as described previously.

### CD19 Co-culture with drug treatments / blocking antibodies

Z-VAD-FMK (Caymen Chemical), Z-IETD-FMK (Thermo Fisher Scientific), 3,4-dichloroisocoumarin (DCI, Santa Cruz Biotechnology; Dallas, TX USA), anti-Fas antibody (Thermo Fisher Scientific), anti-TRAIL antibody (Biolegend), anti-TNFα antibody (Cell Signaling Technology, Danvers, MA USA), and anti-IFNγ antibody (Thermo Fisher Scientific) were purchased from the indicated vendors. For caspase inhibition experiments, Z-VAD-FMK and Z-IETD-FMK were added to target cells 1 h prior to c-culture. For DCI experiments, DCI was added to T cells 1 h prior to co-culture. For blocking antibody experiments, blocking antibodies were added to target cells 1 h prior to co-culture.

### Granzyme B Fluorimetric Assay

Recombinant granzyme B (provided in NOVUS Biologicals assay kit; Centennial, CO USA) was incubated with DCI and other granzyme inhibitors for 30 min at room temperature. The assay was performed on these samples according to the manufacturer’s instructions. Fluorescence was read on a TECAN instrument.

### Conditioned media experiments

Conditioned media was harvested at 24 h following CAR T co-culture. Supernatant from CAR T co-culture was collected and pooled, then spun at 500 rcf for 5 min. The supernatant was passed through a 0.2 µm filter and the conditioned media was then either used immediately or stored at −20 °C for long term use. Target cells were seeded at 5,000 cells / well in 100 µL media prior to conditioned media treatment. For conditioned media viability assays, seeding media was aspirated and cells were incubated with 180 µL of conditioned media for 24 h. Annexin V / Hoechst viability staining was then performed as described previously.

### Cytokine array / Cytokine array XL (R&D Systems, Minneapolis, MN USA)

Conditioned media was collected as described above. 1 mL conditioned media was incubated with cytokine array membranes according to the manufacturer’s instructions overnight at 4 °C. The following day, membranes were washed and incubated with a secondary antibody according to the manufacturer’s instructions. The arrays were quantified using an LASImageQuant4000. Densitometry analysis was performed using GelQuant.NET software provided by biochemlabsolutions.com.

### Colony formation assay

Target cells were seeded as described previously one day prior to CAR T co-culture (day 0). On day 1, CAR T cells were added to the target cells, with some wells receiving only media as a negative control. On day 2, 24 h after CAR-T co-culture, target cells were trypsinized and harvested. Harvested cells from the “no T cell” negative control condition were counted and plated at a final concentration of 200 cells / mL in a 24 well plate. The co-cultured target cells at all E:T ratios were diluted to the same ratio as the “no T cell” control. Cells were left undisturbed in an incubator at 37 °C for six days, at which time they were washed with HBSS and fixed with 10% neutral buffered formalin. After fixation, the cells were stained with crystal violet and allowed to dry overnight. Colonies were defined as clusters of 4 cells or more, and were quantified by eye using a light microscope.

### Generation of stable Bcl-2 and Bcl-XL expressing JeKo-1 and Nalm6 variants

pCDH-puro-Bcl-XL and pCDH-puro-Bcl2 were gifts from Jialiang Wang (Addgene plasmid #46972 and #46971). psPAX2 was a gift from Didier Trono (Addgene plasmid #12260) and pCI-VSVG was a gift from Garry Nolan (Addgene plasmid #1733). The pCDH-empty vector construct was created by removing the Bcl-2 insert from the pCDH-puro-Bcl2 plasmid via digestion with EcoRI. This new plasmid, designated “pCDH-empty”, was transfected into XL10-Gold competent cells according to the manufacturer’s instruction (Agilent technologies / STRATAGENE, Santa Clara, CA USA). DNA was purified from bacteria using a Qiagen MIDI Prep kit according to the manufacturer’s instructions (Hilden, Germany).

### Lentivirus generation and harvesting

HEK 293T/17 cells were transfected with pCDH-puro-Bcl2, pCDH-puro-Bcl-XL, and pCDH-puro-empty plasmids in combination with pCI-VSVG and and psPax2 to generate lentivirus. Viral supernatant was harvested at 24 h and 48 h, pooled, and frozen at −80 °C. Viral titer / MOI was estimated using the QUICKTITER p24 ELISA KIT from Cell BioLabs Inc (San Diego, CA USA), which was performed according to the manufacturer’s instructions.

### Transduction of JeKo-1 and Nalm6 cells

JeKo-1 cells were incubated with polybrene and lentivirus at multiple MOIs. 48 h after transduction, cells were centrifuged and placed in fresh RPMI-10%FBS medium with puromycin. Puromycin selection (1 µg/mL) continued for 14 days while cells were expanded. Following selection, cells were maintained in the appropriate media with FBS, PenStrep, plasmocin, and 0.5 µg/mL puromycin.

### JeKo-1 and Nalm6 variant co-culture with CD19 CAR T cells

One day prior to CAR T co-culture, target cells were labeled with CFDA-SE. On the day of co-culture, target cells and CAR T cells at varying cell numbers were incubated together in a 96 well plate at the E:T ratios indicated. 24 h following co-culture, the cells were collected and stained with annexin V / Hoechst as described previously.

### CellTiter-Glo viability assay

JeKo-1 cells were plated in 384 well plates for CellTiter-Glo (Promega; Madison, WI USA) experiments at a density of 7000 cells / well in 30 µL. 15 µL of the indicated treatment was added to the plate. Viability was assessed at 24, 48, or 72 h via the addition of 15 µL CellTiter-Glo reagent. Cells were incubated with reagent and protected from light for 10 min prior to luminescence quantification on a Magellan TECAN. Results are presented normalized to luminescence in the negative control wells.

### Statistical analysis

The goal of statistical tests presented in this work was to compare differences in CAR T killing between different target cell populations. Statistical analysis was performed using GraphPad Prism 9 software. Unless otherwise indicated, killing was estimated by the E:T50 or IC50 value, which was approximated from curve fitting as described above. Statistical comparison of mean E:T50 or IC50 values was accomplished by unpaired, one-tailed *t* tests, generated from the following data: mean = logIC50 for each curve; error = standard error of logIC50; *N*=(1+degree of freedom for the curve fit). When multiple comparisons were performed, the *p* value threshold of 0.05 was Bonferroni corrected to 0.025 (for two comparisons) or 0.0167 (for three comparisons). For the paired experiments presented in Figs. 2D, 6C, and 6E, statistical comparison of E:T 50 values was accomplished by paired, one-tailed *t* tests, generated from the following data: mean = logIC50 for each curve; error = standard error of logIC50; *N*=(1+degree of freedom for the curve fit).

## Supplementary information

Supplemental Figure 1

Supplemental Figure 2

Supplemental Figure 3

Supplemental Figure 4

Supplemental Figure 5

Supplemental Figure 6

Supplemental Figure 7

Supplemental Figure 8

Supplemental Figure 9

Supplemental Figure 10

Supplemental Figure 11

Supplemental Figure 12

Supplemental Table 1

Supplemental Table 2

Supplemental Table 3

Supplemental Table 4

Reproducibility Checklist

## Data Availability

Data sharing is not applicable to this article as no datasets were generated or analyzed during the current study.

## References

[1] Kochenderfer JN, Wilson WH, Janik JE, Dudley ME, Stetler-Stevenson M, Feldman SA (2010). Eradication of B-lineage cells and regression of lymphoma in a patient treated with autologous T cells genetically engineered to recognize CD19. Blood..

[2] Kalos M, Levine BL, Porter DL, Katz S, Grupp SA, Bagg A (2011). T cells with chimeric antigen receptors have potent antitumor effects and can establish memory in patients with advanced leukemia. Sci Transl Med.

[3] McHayleh W, Bedi P, Sehgal R, Solh M (2019). Chimeric antigen receptor T-cells: the future is now. J Clin Med.

[4] Maus MV, June CH (2016). Making better chimeric antigen receptors for adoptive T-cell therapy. Clin Cancer Res.

[5] Almasbak H, Aarvak T, Vemuri MC (2016). CAR T cell therapy: a game changer in cancer treatment. J Immunol Res.

[6] Oluwole OO, Davila ML (2016). At The Bedside: clinical review of chimeric antigen receptor (CAR) T cell therapy for B cell malignancies. J Leukoc Biol.

[7] Ni Chonghaile T, Sarosiek KA, Vo TT, Ryan JA, Tammareddi A, Moore Vdel G (2011). Pretreatment mitochondrial priming correlates with clinical response to cytotoxic chemotherapy. Science..

[8] Montero J, Sarosiek KA, DeAngelo JD, Maertens O, Ryan J, Ercan D (2015). Drug-induced death signaling strategy rapidly predicts cancer response to chemotherapy. Cell..

[9] Warren CFA, Wong-Brown MW, Bowden NA (2019). BCL-2 family isoforms in apoptosis and cancer. Cell Death Dis.

[10] Yip KW, Reed JC (2008). Bcl-2 family proteins and cancer. Oncogene..

[11] Perini GF, Ribeiro GN, Pinto Neto JV, Campos LT, Hamerschlak N (2018). BCL-2 as therapeutic target for hematological malignancies. J Hematol Oncol.

[12] Vo TT, Letai A (2010). BH3-only proteins and their effects on cancer. Adv Exp Med Biol.

[13] Green DR, Llambi F (2015). Cell death signaling. Cold Spring Harb Perspect Biol.

[14] Kale J, Osterlund EJ, Andrews DW (2018). BCL-2 family proteins: changing partners in the dance towards death. Cell Death Differ.

[15] Kalkavan H, Green DR (2018). MOMP, cell suicide as a BCL-2 family business. Cell Death Differ.

[16] Zamaraeva MV, Sabirov RZ, Maeno E, Ando-Akatsuka Y, Bessonova SV, Okada Y (2005). Cells die with increased cytosolic ATP during apoptosis: a bioluminescence study with intracellular luciferase. Cell Death Differ.

[17] Kantari C, Walczak H (2011). Caspase-8 and bid: caught in the act between death receptors and mitochondria. Biochim Biophys Acta.

[18] Jost PJ, Grabow S, Gray D, McKenzie MD, Nachbur U, Huang DC (2009). XIAP discriminates between type I and type II FAS-induced apoptosis. Nature..

[19] Kaiserman D, Bird CH, Sun J, Matthews A, Ung K, Whisstock JC (2006). The major human and mouse granzymes are structurally and functionally divergent. J Cell Biol.

[20] Casciola-Rosen L, Garcia-Calvo M, Bull HG, Becker JW, Hines T, Thornberry NA (2007). Mouse and human granzyme B have distinct tetrapeptide specificities and abilities to recruit the bid pathway. J Biol Chem.

[21] Pinkoski MJ, Waterhouse NJ, Heibein JA, Wolf BB, Kuwana T, Goldstein JC (2001). Granzyme B-mediated apoptosis proceeds predominantly through a Bcl-2-inhibitable mitochondrial pathway. J Biol Chem.

[22] Heibein JA, Goping IS, Barry M, Pinkoski MJ, Shore GC, Green DR (2000). Granzyme B-mediated cytochrome c release is regulated by the Bcl-2 family members bid and Bax. J Exp Med.

[23] Barry M, Heibein JA, Pinkoski MJ, Lee SF, Moyer RW, Green DR (2000). Granzyme B short-circuits the need for caspase 8 activity during granule-mediated cytotoxic T-lymphocyte killing by directly cleaving Bid. Mol Cell Biol.

[24] Ravi R, Fuchs EJ, Jain A, Pham V, Yoshimura K, Prouser T (2006). Resistance of cancers to immunologic cytotoxicity and adoptive immunotherapy via X-linked inhibitor of apoptosis protein expression and coexisting defects in mitochondrial death signaling. Cancer Res.

[25] Jaime-Sanchez P, Catalan E, Uranga-Murillo I, Aguilo N, Santiago L, ML P (2018). Antigen-specific primed cytotoxic T cells eliminate tumour cells in vivo and prevent tumour development, regardless of the presence of anti-apoptotic mutations conferring drug resistance. Cell Death Differ.

[26] Pardo J, Wallich R, Martin P, Urban C, Rongvaux A, Flavell RA (2008). Granzyme B-induced cell death exerted by ex vivo CTL: discriminating requirements for cell death and some of its signs. Cell Death Differ.

[27] Singh N, Lee YG, Shestova O, Ravikumar P, Hayer KE, Hong SJ (2020). Impaired death receptor signaling in leukemia causes antigen-independent resistance by inducing CAR T cell dysfunction. Cancer Discov.

[28] Karlsson H, Lindqvist AC, Fransson M, Paul-Wetterberg G, Nilsson B, Essand M (2013). Combining CAR T cells and the Bcl-2 family apoptosis inhibitor ABT-737 for treating B-cell malignancy. Cancer Gene Ther.

[29] Yan X, Chen D, Wang Y, Guo Y, Tong C, Wei J (2022). Identification of NOXA as a pivotal regulator of resistance to CAR T-cell therapy in B-cell malignancies. Signal Transduct Target Ther.

[30] Koss B, Ryan J, Budhraja A, Szarama K, Yang X, Bathina M (2016). Defining specificity and on-target activity of BH3-mimetics using engineered B-ALL cell lines. Oncotarget..

[31] Ryan J, Montero J, Rocco J, Letai A (2016). iBH3: simple, fixable BH3 profiling to determine apoptotic priming in primary tissue by flow cytometry. Biol Chem.

[32] Boroughs AC, Larson RC, Marjanovic ND, Gosik K, Castano AP, Porter CBM (2020). A distinct transcriptional program in human CAR T cells bearing the 4-1BB signaling domain revealed by scRNA-Seq. Mol Ther.

[33] Wang W, Green M, Choi JE, Gijon M, Kennedy PD, Johnson JK (2019). CD8(+) T cells regulate tumour ferroptosis during cancer immunotherapy. Nature..

[34] Zhang Z, Zhang Y, Xia S, Kong Q, Li S, Liu X (2020). Gasdermin E suppresses tumour growth by activating anti-tumour immunity. Nature..

[35] Ormhoj M, Scarfo I, Cabral ML, Bailey SR, Lorrey SJ, Bouffard AA (2019). Chimeric antigen receptor T cells targeting CD79b show efficacy in lymphoma with or without cotargeting CD19. Clin Cancer Res.

