# Supplementary material for: Quantifying requirements for mitochondrial apoptosis in CAR T killing of cancer cells
Source: Cell Death Dis. 2023 Apr 13;14(4):267. doi: 10.1038/s41419-023-05727-x (PMC10101951; doi:10.1038/s41419-023-05727-x)
Supplement: Supplementary file 1 — Supplemental Figure 1 [file 41419_2023_5727_MOESM1_ESM.pdf]

A

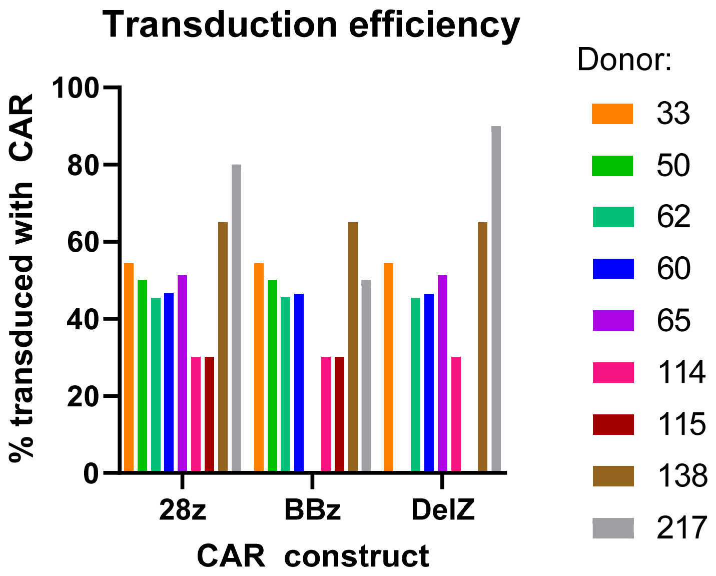

B

Specific killing achieved?		
Donor # + target	Costimulatory Domain	
	28z	BBz
31 CD19	Yes	No
33 CD19	Yes	Yes
50 CD19	Yes	No
62 CD19	Yes	Yes
60 CD19	Yes	Yes
65 CD19	Yes	*Not tested*
114 CD19	Yes	Yes
115 CD19	No	Yes
138 CD19	Yes	Yes
217 CD19	Yes	Yes
217 EGFR	Yes	No
218 EGFR	Yes	No

Figure S1.
A) Transduction efficiency of CD19-directed CARs, sorted by donor, used in this manuscript. **B)** Comparison of specific killing (killing above negative control CAR) for both costimulatory domains across all donors tested. There were five instances where a certain costimulatory domain failed to achieve specific killing above the level of the negative control. These CAR T cells were excluded from further analysis.
