# Supplementary material for: Quantifying requirements for mitochondrial apoptosis in CAR T killing of cancer cells
Source: Cell Death Dis. 2023 Apr 13;14(4):267. doi: 10.1038/s41419-023-05727-x (PMC10101951; doi:10.1038/s41419-023-05727-x)
Supplement: Supplementary file 2 — Supplemental Figure 2 [file 41419_2023_5727_MOESM2_ESM.pdf]

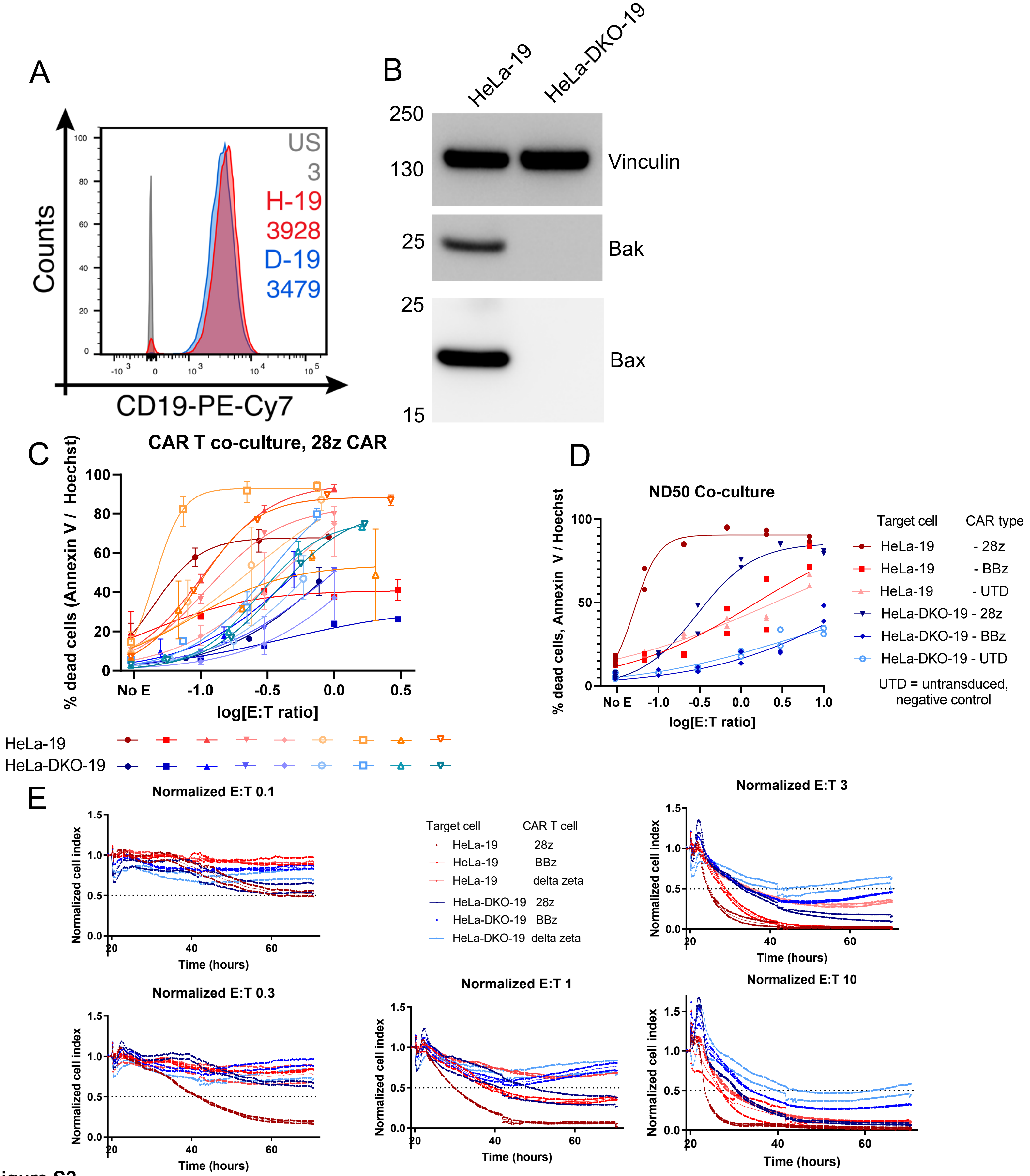


**Figure S2.**

**A**) CD19 staining intensity as measured by flow cytometry, US=unstained, H-19 = HeLa-19, D-19 = HeLa-DKO-19. **B**) Immunoblotting for Bak and Bax in HeLa-19 and HeLa-DKO-19 cell lines. **C**) Demonstration of E:T 50 estimation presented in Figure 2C with representative experiment, see Table S1 for values. **D**) All nine paired biological replicates from a single donor's CAR T cells, including those presented in Figure 2C. Error bars depict range of technical replicates. **E)** Normalized impedance over time, as measured on an Acea xCELLigence. All curves are normalized to either HeLa-19 cells alone or HeLa-DKO-19 cells alone. CAR T cells were added at ~19 hours, and the measurements concluded at 72 hours. Reduced cell index corresponds to reduced impedance, indicating cell death. These are representative images from one of two independent experiments.
