# Supplementary material for: Quantifying requirements for mitochondrial apoptosis in CAR T killing of cancer cells
Source: Cell Death Dis. 2023 Apr 13;14(4):267. doi: 10.1038/s41419-023-05727-x (PMC10101951; doi:10.1038/s41419-023-05727-x)
Supplement: Supplementary file 3 — Supplemental Figure 3 [file 41419_2023_5727_MOESM3_ESM.pdf]

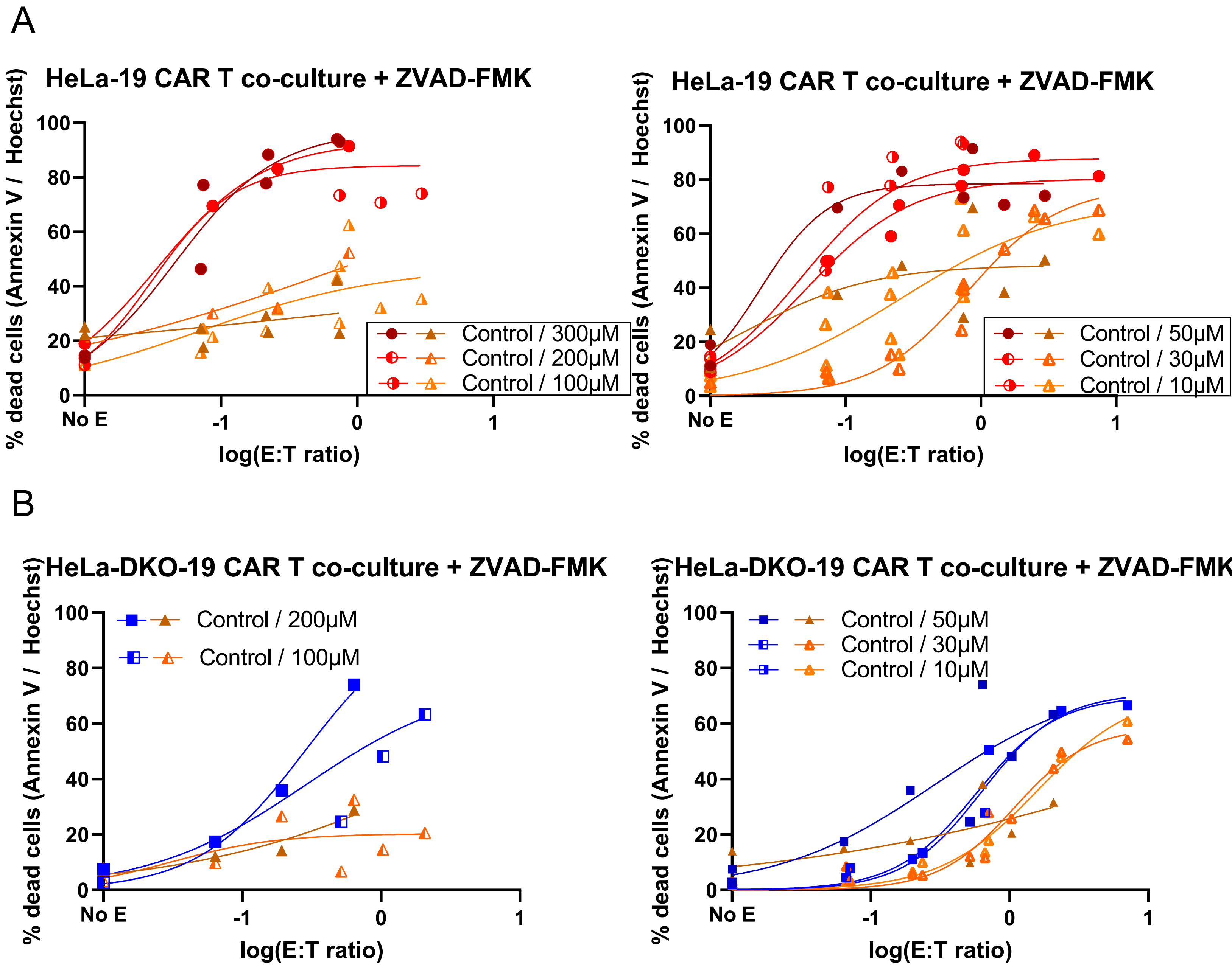

Figure S3.
Annexin V / Hoechst viability staining following CAR T co-culture of (**A**) HeLa-19 target cells or (**B**) HeLa-DKO-19 target cells with additional doses of the caspase inhibitor Z-VAD-FMK.
