# Supplementary material for: Quantifying requirements for mitochondrial apoptosis in CAR T killing of cancer cells
Source: Cell Death Dis. 2023 Apr 13;14(4):267. doi: 10.1038/s41419-023-05727-x (PMC10101951; doi:10.1038/s41419-023-05727-x)
Supplement: Supplementary file 4 — Supplemental Figure 4 [file 41419_2023_5727_MOESM4_ESM.pdf]

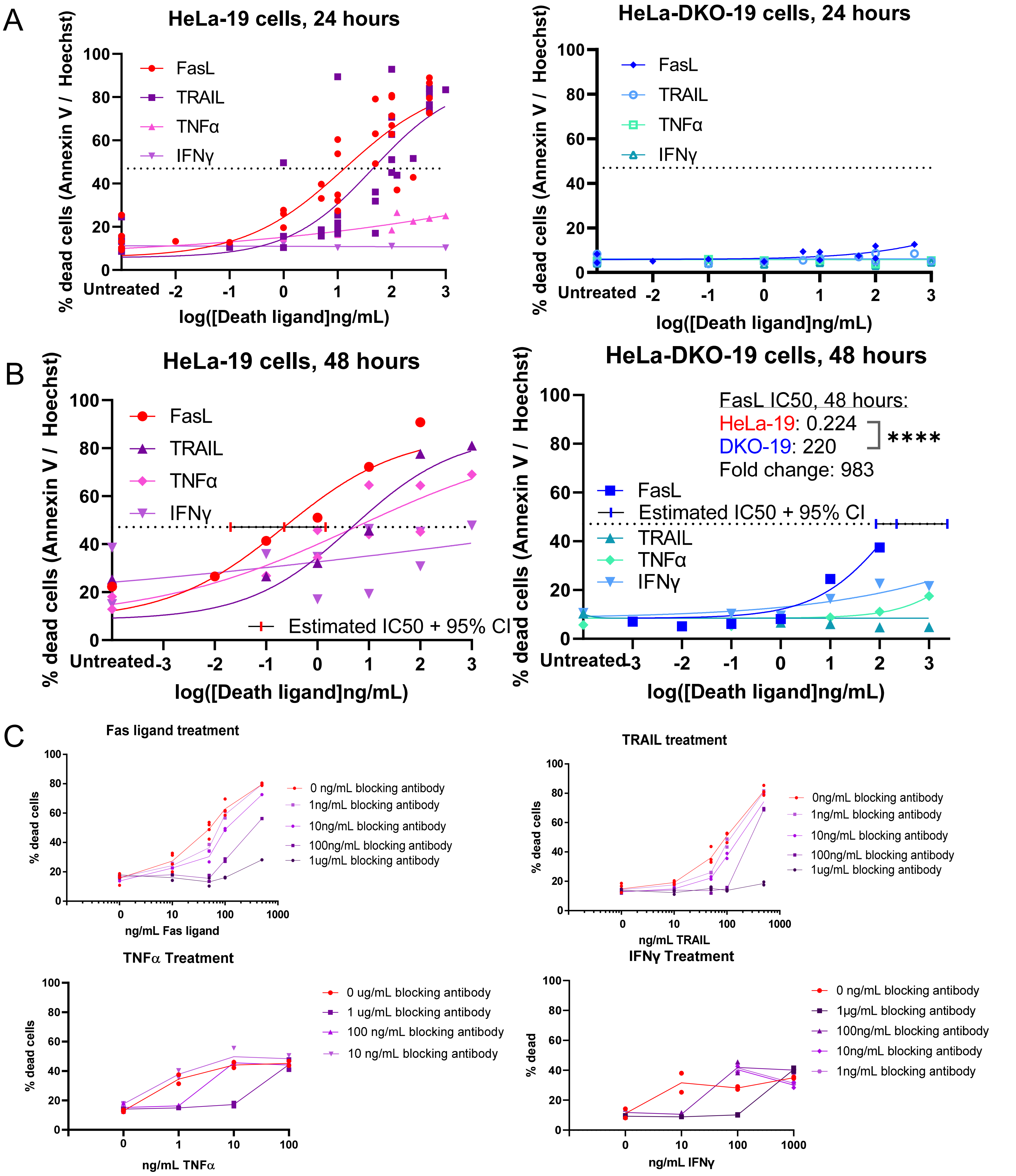

Figure S4.

Annexin V / Hoechst viability staining at 24 hours (**A**) or 48 hours (**B**) following treatment with the recombinant death ligand or cytokine at the indicated dose. Each point is a biological replicate, **** indicates p<0.0001, unpaired t test. (**C**) Annexin V / Hoechst viability staining of HeLa-19 cells following 24 hours (FasL, TRAIL) or 48 hours (TNFα, IFNγ) of treatment with recombinant death ligand or cytokine and the indicated dose of blocking antibody. Representative experiment shown, each point is a technical replicate.
