# Supplementary material for: Quantifying requirements for mitochondrial apoptosis in CAR T killing of cancer cells
Source: Cell Death Dis. 2023 Apr 13;14(4):267. doi: 10.1038/s41419-023-05727-x (PMC10101951; doi:10.1038/s41419-023-05727-x)
Supplement: Supplementary file 5 — Supplemental Figure 5 [file 41419_2023_5727_MOESM5_ESM.pdf]

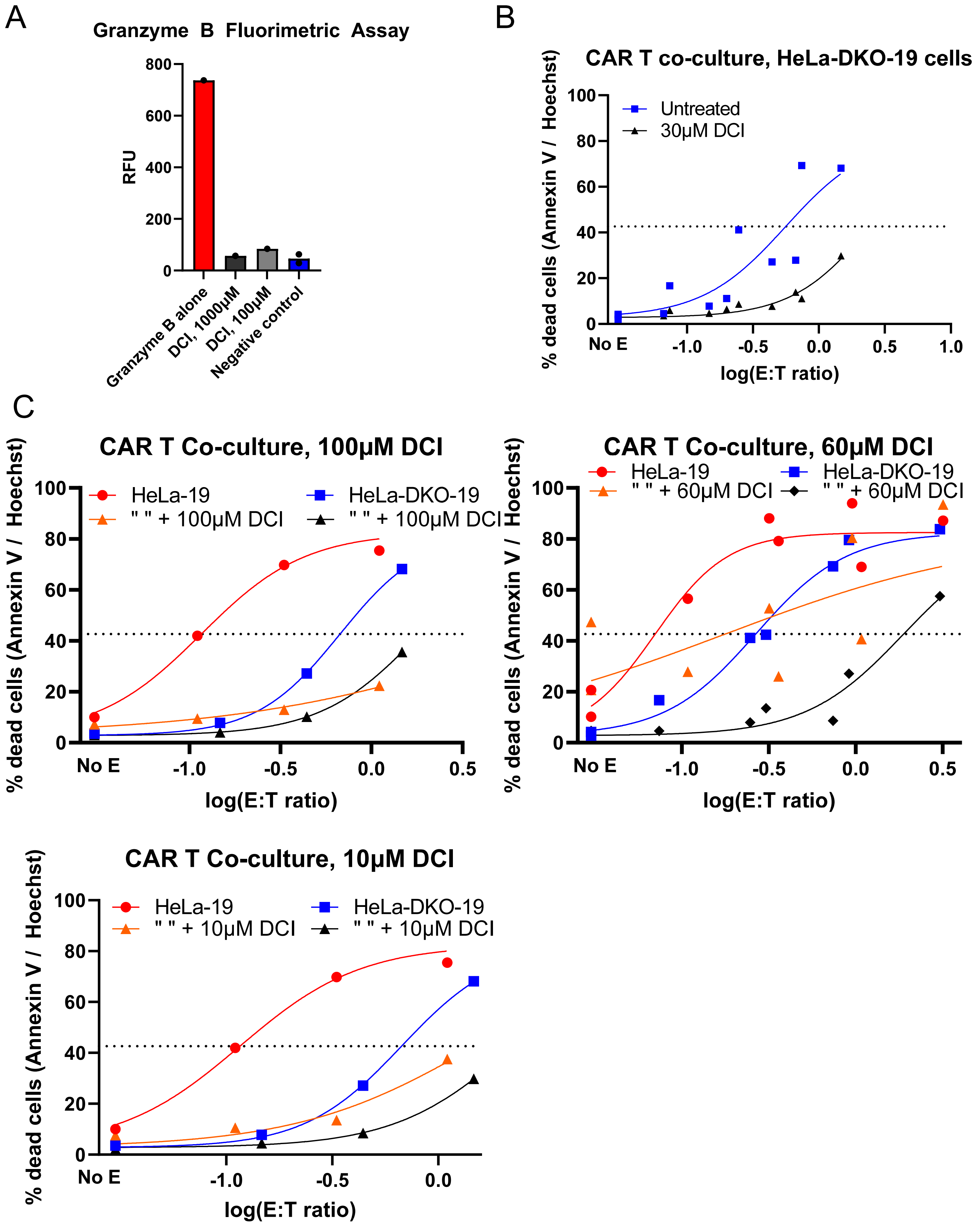

Figure S5.

A) Fluorimetric granzyme B assay comparing recombinant granzyme B activity in the presence of 3,4 dichloroisocoumarin (DCI). N = 1, each point is a technical duplicate. **B**) HeLa-DKO-19 viability following CAR T co-culture with T cells pretreated with 30μDCI. **C**) HeLa-19 and HeLa-DKO-19 target cell viability following co-culture with CAR T cells following T cell pre-treatment with the indicated doses of DCI. Each point is a biological replicate.
