# Supplementary material for: Quantifying requirements for mitochondrial apoptosis in CAR T killing of cancer cells
Source: Cell Death Dis. 2023 Apr 13;14(4):267. doi: 10.1038/s41419-023-05727-x (PMC10101951; doi:10.1038/s41419-023-05727-x)
Supplement: Supplementary file 6 — Supplemental Figure 6 [file 41419_2023_5727_MOESM6_ESM.pdf]

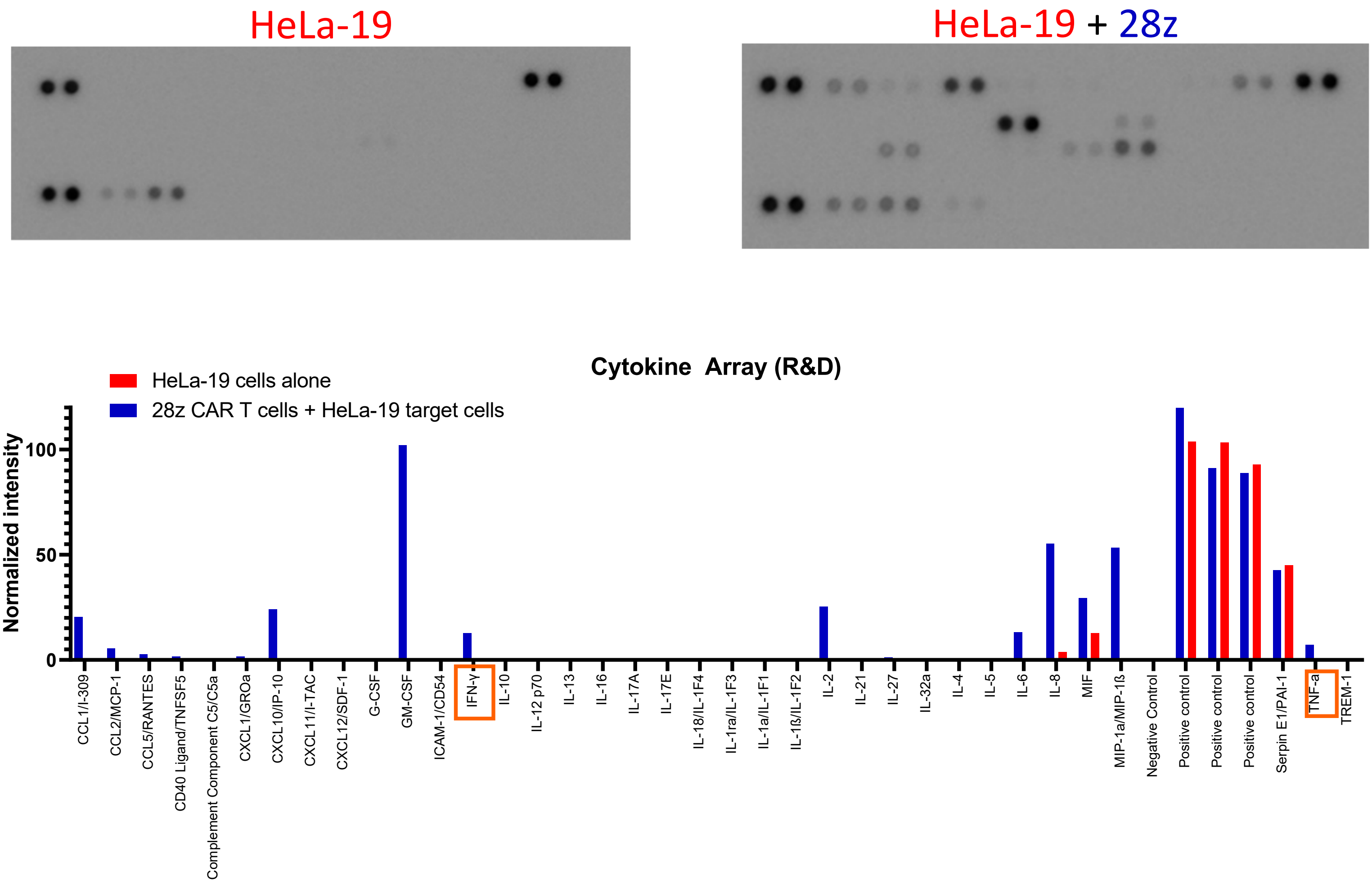

Figure S6.
Cytokine Array: Raw data (top) and graphed normalized intensity values (bottom) for cytokine array presented in Figure 5E. See list of results in Table S2.
