# Supplementary material for: Quantifying requirements for mitochondrial apoptosis in CAR T killing of cancer cells
Source: Cell Death Dis. 2023 Apr 13;14(4):267. doi: 10.1038/s41419-023-05727-x (PMC10101951; doi:10.1038/s41419-023-05727-x)
Supplement: Supplementary file 8 — Supplemental Figure 8 [file 41419_2023_5727_MOESM8_ESM.pdf]

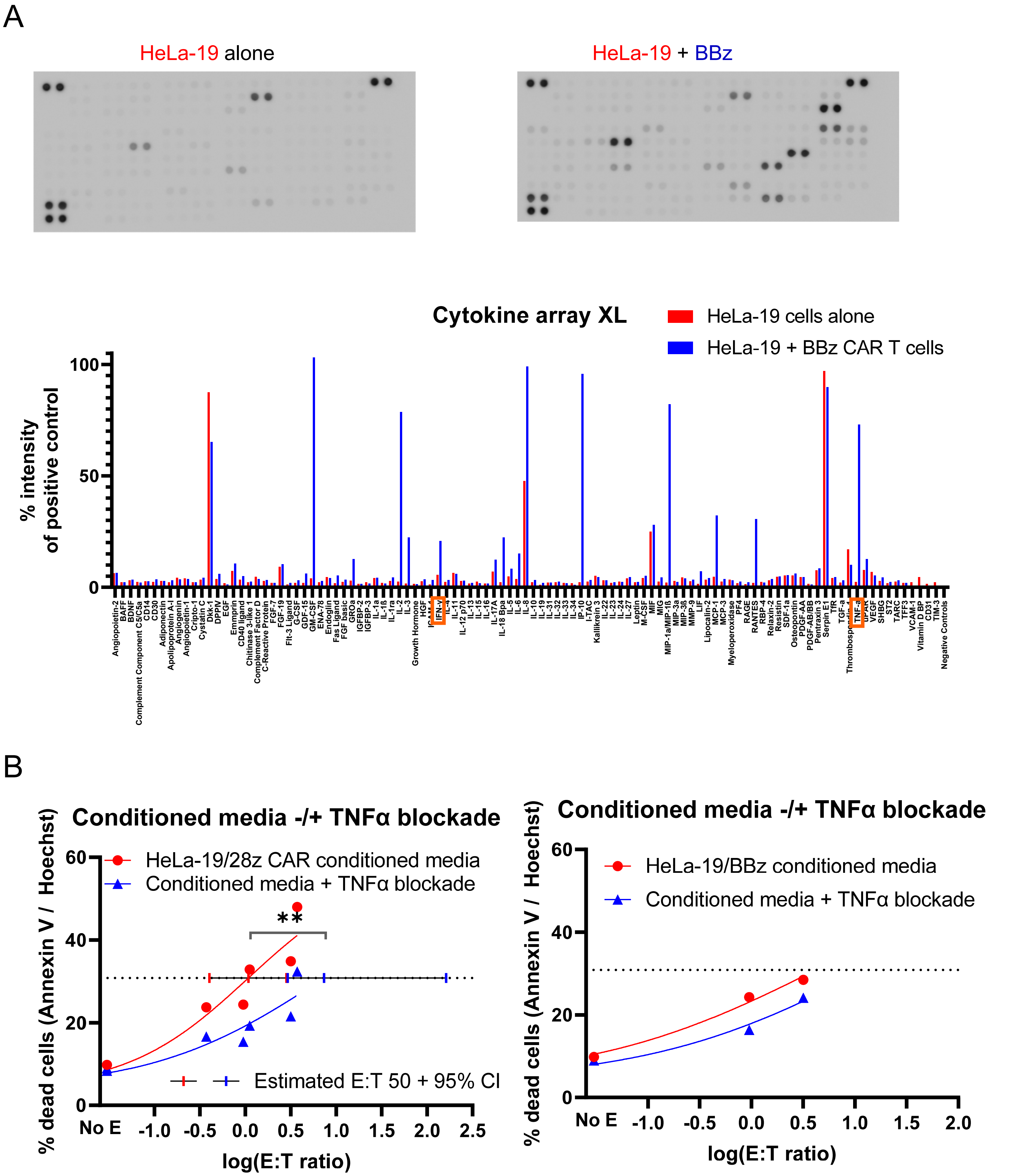

Figure S8.
A. Cytokine Array XL. Raw data (top panel) and graphed normalized intensity values (bottom panel) from cytokine array presented in Figure 5E. Normalized intensity values are presented in Table S4. **B.** Annexin V / Hoechst viability assay of HeLa-19 target cells exposed to conditioned media from 28z CAR T co-culture (left panel, N=2) or BBz CAR T co-culture (right panel, N=1). These data are presented together in Figure 5F. Each point is a biological replicate, ** indicates p = 0.0083, unpaired t test.
