# Supplementary material for: Quantifying requirements for mitochondrial apoptosis in CAR T killing of cancer cells
Source: Cell Death Dis. 2023 Apr 13;14(4):267. doi: 10.1038/s41419-023-05727-x (PMC10101951; doi:10.1038/s41419-023-05727-x)
Supplement: Supplementary file 9 — Supplemental Figure 9 [file 41419_2023_5727_MOESM9_ESM.pdf]

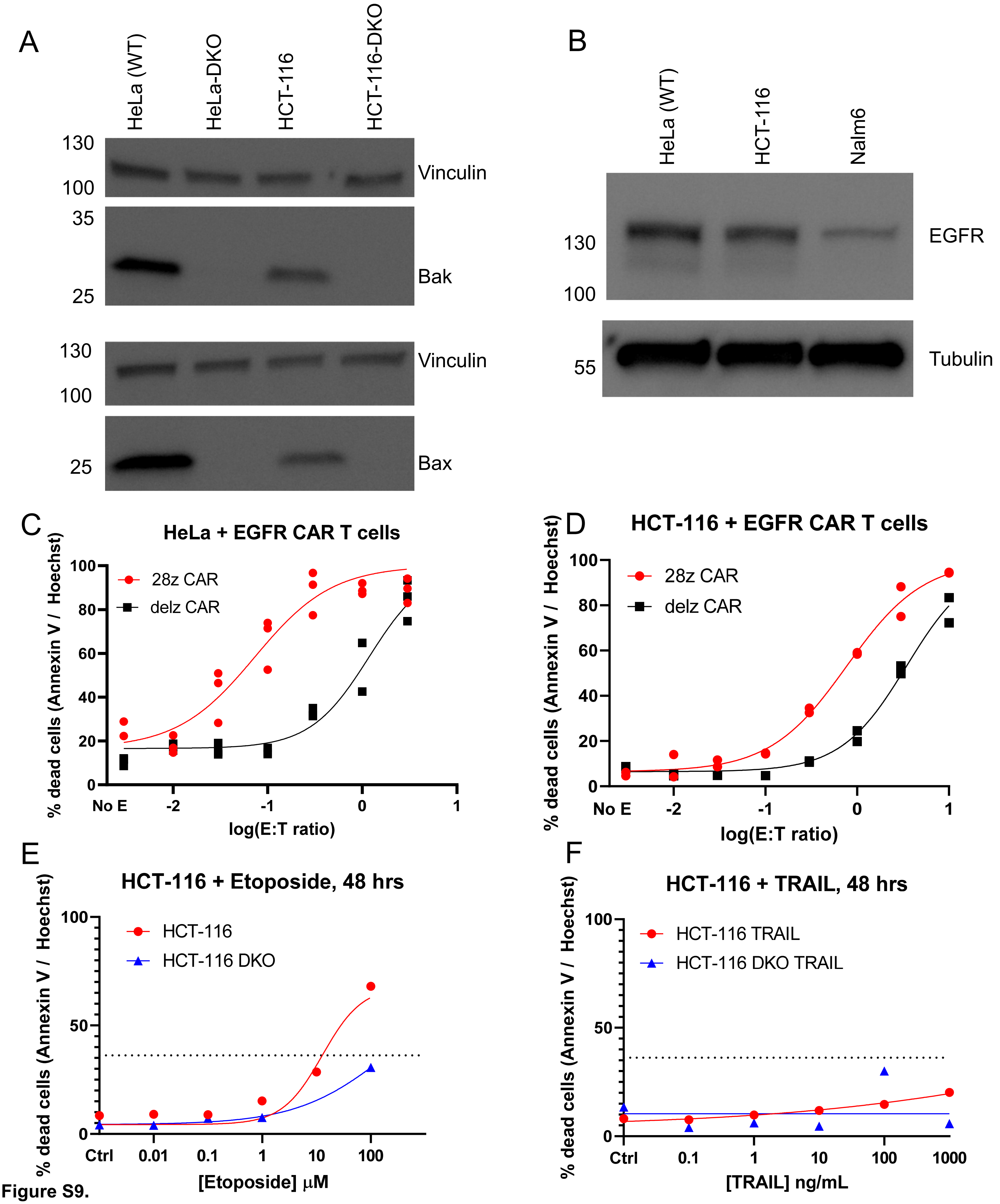

Figure S9.
A) Immunoblotting Bak, Bax, and vinculin (loading control) in HeLa and HCT-116 wildtype and isogenic Bak/Bax knockout cell lines. **B**) Immunoblotting for EGFR and tubulin (loading control) in HeLa, HCT-116, and Nalm6 cell lines. **C-D**) 24 hour Annexin V / Hoechst viability data following HeLa (**C**) and HCT-116 (**D**) co-culture with specific (28z) and negative control (delta zeta) CAR T cells recognizing EGFR, N=1, each point is a technical replicate. **D-E**) 48 hour Annexin V / Hoechst viability following HCT-116 and HCT-116-DKO cell line treatment with etoposide or TRAIL at the indicated doses. N=1.
