# Supplementary material for: Quantifying requirements for mitochondrial apoptosis in CAR T killing of cancer cells
Source: Cell Death Dis. 2023 Apr 13;14(4):267. doi: 10.1038/s41419-023-05727-x (PMC10101951; doi:10.1038/s41419-023-05727-x)
Supplement: Supplementary file 10 — Supplemental Figure 10 [file 41419_2023_5727_MOESM10_ESM.pdf]

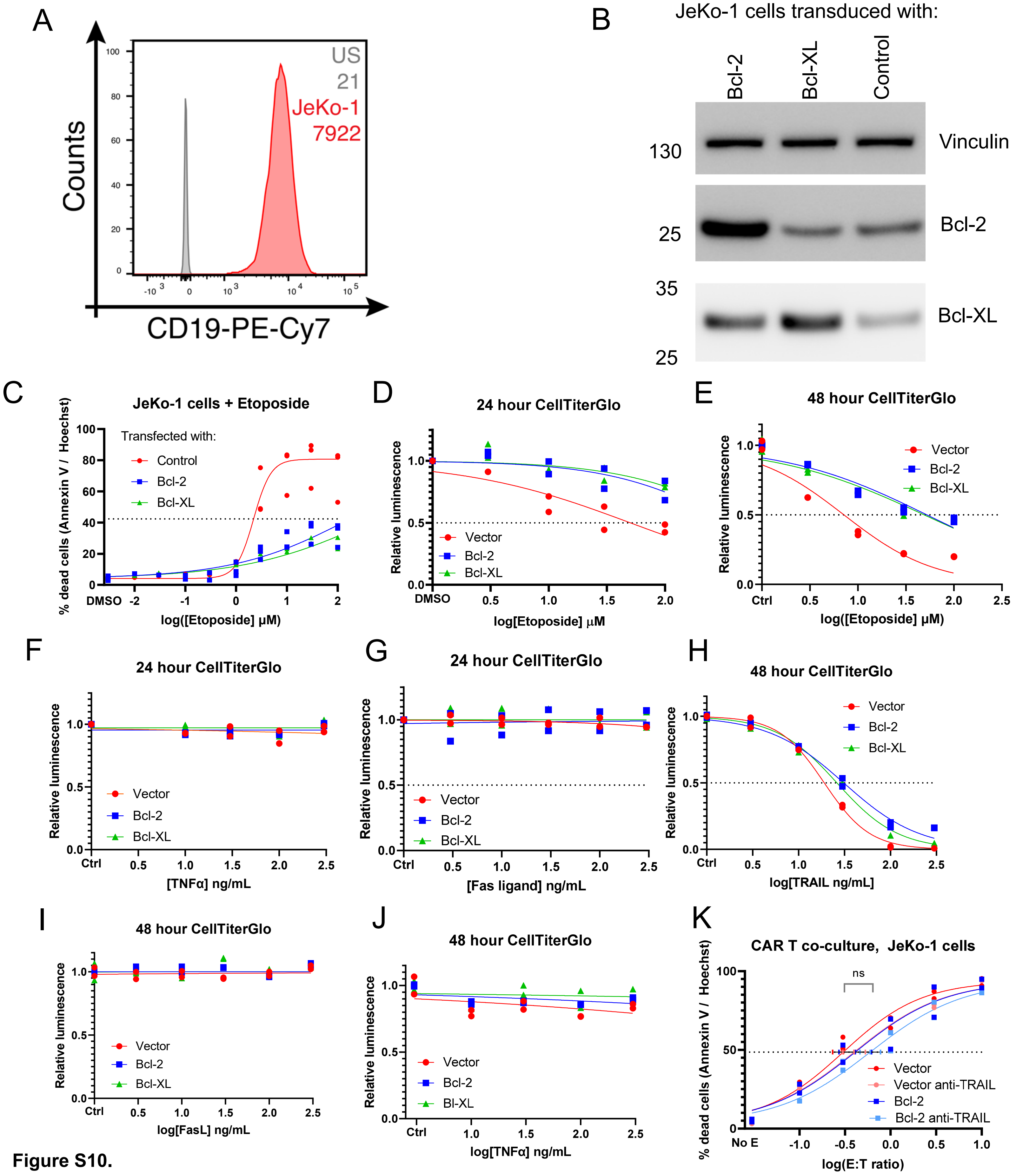

Figure S10.

A) CD19 expression in JeKo-1 cells via flow cytometry. **B**) Immunoblotting for Bcl-2 and Bcl-XL in JeKo-1 cells transduced with empty vector control, Bcl-2, or Bcl-XL constructs. **C**) 24 hour Annexin V / Hoechst viability assay following etoposide treatment in each of the JeKo-1 cell lines, N=3, each point is a biological replicate. **D-E**) 24 and 48 hour CellTiter-Glo viability of JeKo-1 variants following etoposide treatment. **F-G)** CellTiter-Glo viability data 24 hours following JeKo-1 variant incubation with the indicated recombinant proteins. N=2, each point is a biological replicate. **H-J**) CellTiter-Glo viability data 48 hours following JeKo-1 variant incubation with the indicated recombinant proteins. N=1, each point is a technical replicate.
