# Supplementary material for: Quantifying requirements for mitochondrial apoptosis in CAR T killing of cancer cells
Source: Cell Death Dis. 2023 Apr 13;14(4):267. doi: 10.1038/s41419-023-05727-x (PMC10101951; doi:10.1038/s41419-023-05727-x)
Supplement: Supplementary file 11 — Supplemental Figure 11 [file 41419_2023_5727_MOESM11_ESM.pdf]

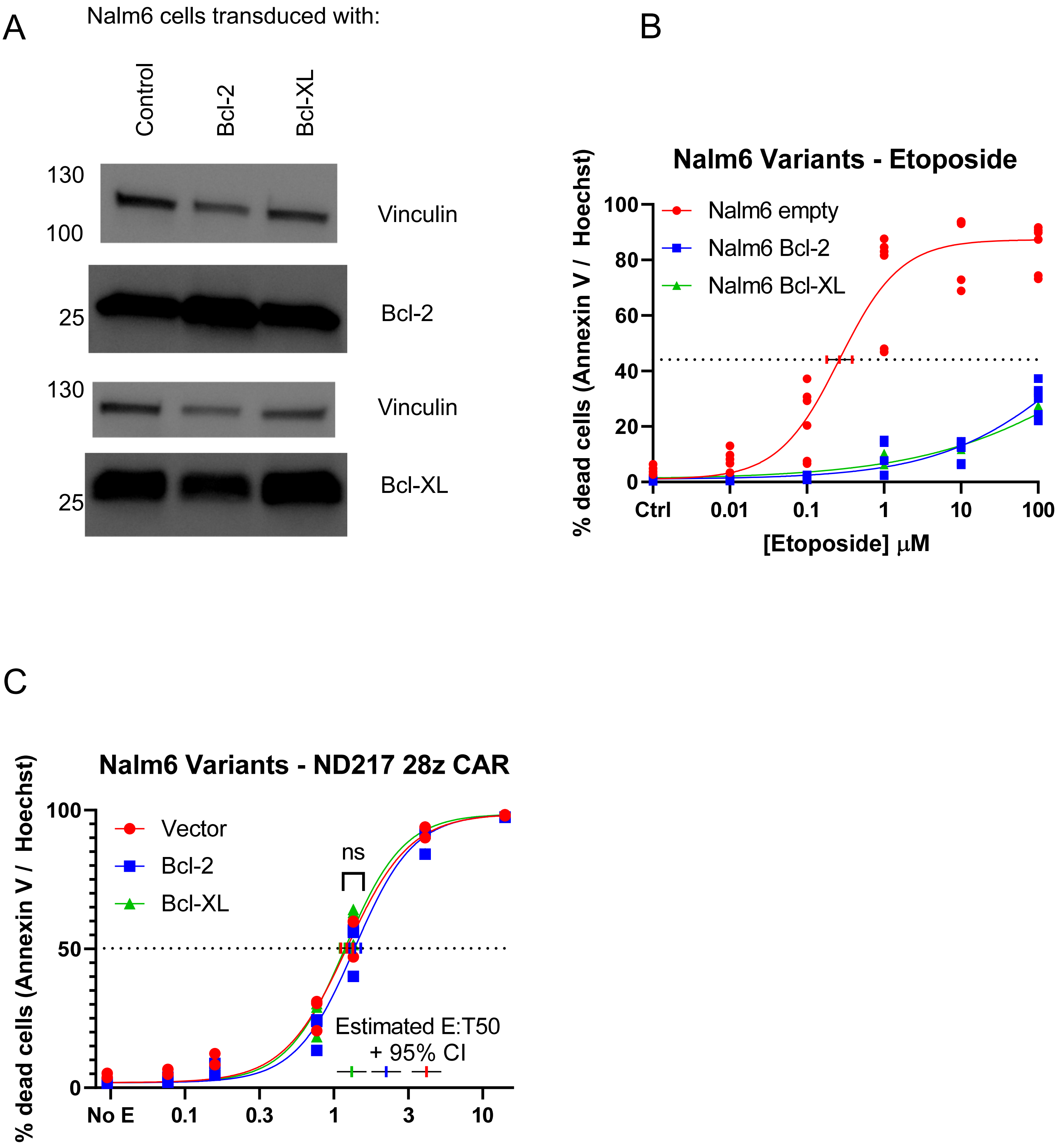

Figure S11.
A) Immunoblotting for Bcl-2 and Bcl-XL in Nalm6 cells transduced with empty vector control, Bcl-2, or Bcl-XL constructs. Vinculin included as a loading control. **B**) 24 hour Annexin V / Hoechst viability assay following etoposide treatment in each of the Nalm6 cell lines, N=3, each point is a biological replicate. **C**) Annexin V / Hoechst viability assay following 24 hour co-culture of CD19-directed CAR T cells and each of the Nalm6 cell lines indicated.
