# Supplementary material for: Quantifying requirements for mitochondrial apoptosis in CAR T killing of cancer cells
Source: Cell Death Dis. 2023 Apr 13;14(4):267. doi: 10.1038/s41419-023-05727-x (PMC10101951; doi:10.1038/s41419-023-05727-x)
Supplement: Supplementary file 12 — Supplemental Figure 12 [file 41419_2023_5727_MOESM12_ESM.pdf]

Figure 1C

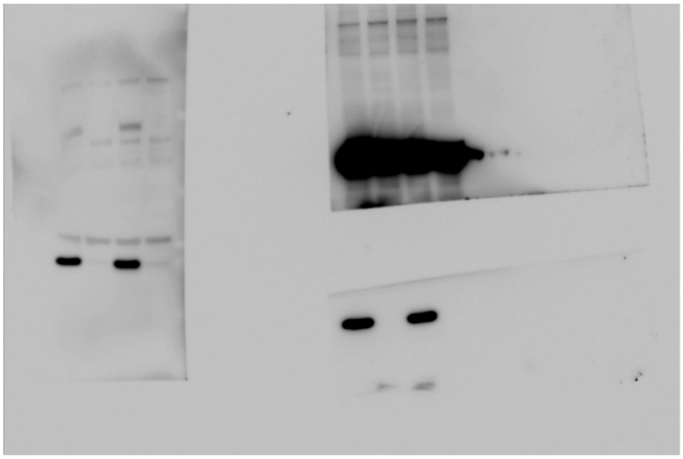

Figure S9A

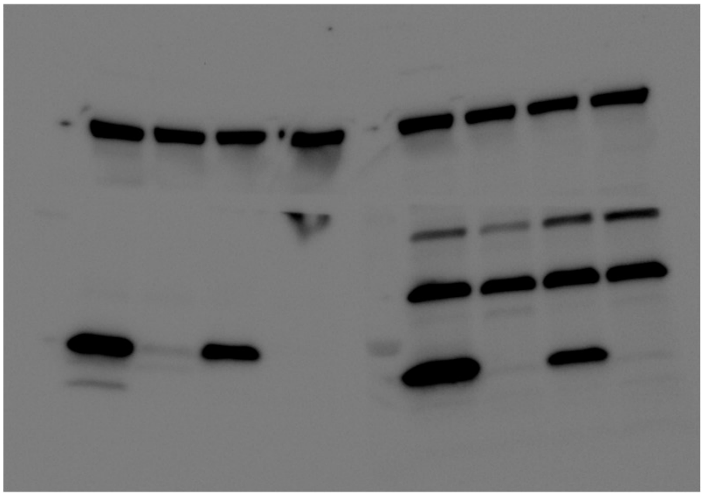

Figure 3A

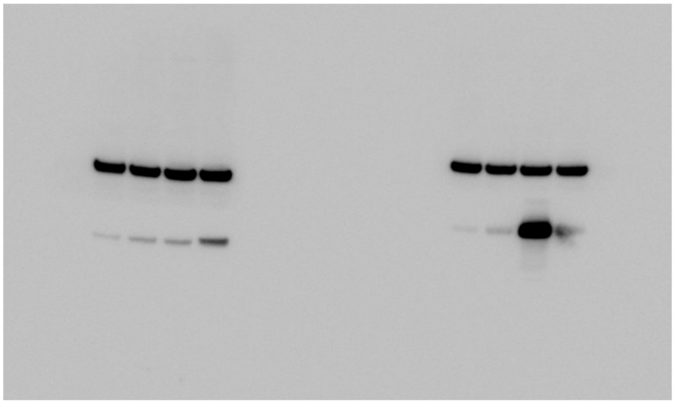

Figure S9B

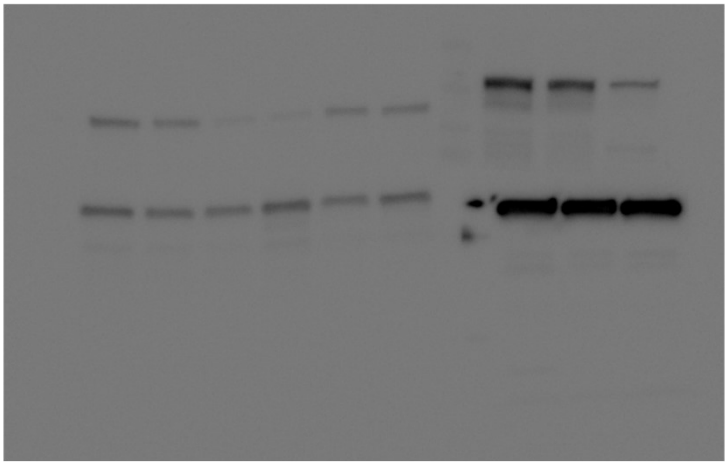

Figure S2B / S10B

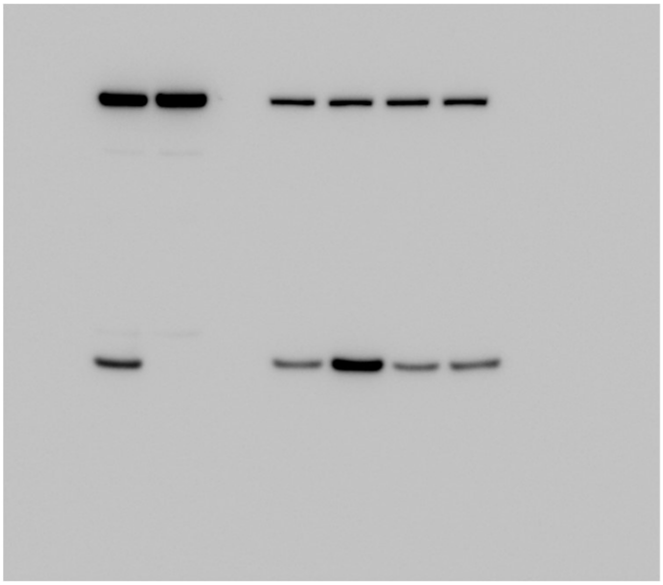

Figure S11A

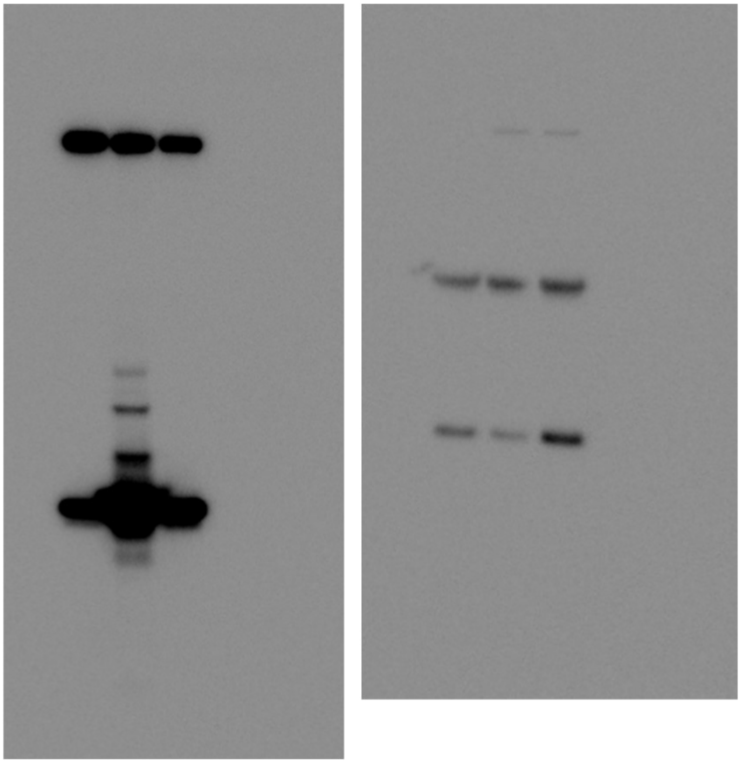

Figure S12.
Uncropped immunoblots from the indicated figures.
