# Supplementary material for: Quantifying requirements for mitochondrial apoptosis in CAR T killing of cancer cells
Source: Cell Death Dis. 2023 Apr 13;14(4):267. doi: 10.1038/s41419-023-05727-x (PMC10101951; doi:10.1038/s41419-023-05727-x)
Supplement: Supplementary file 7 — Supplemental Figure 7 [file 41419_2023_5727_MOESM7_ESM.pdf]

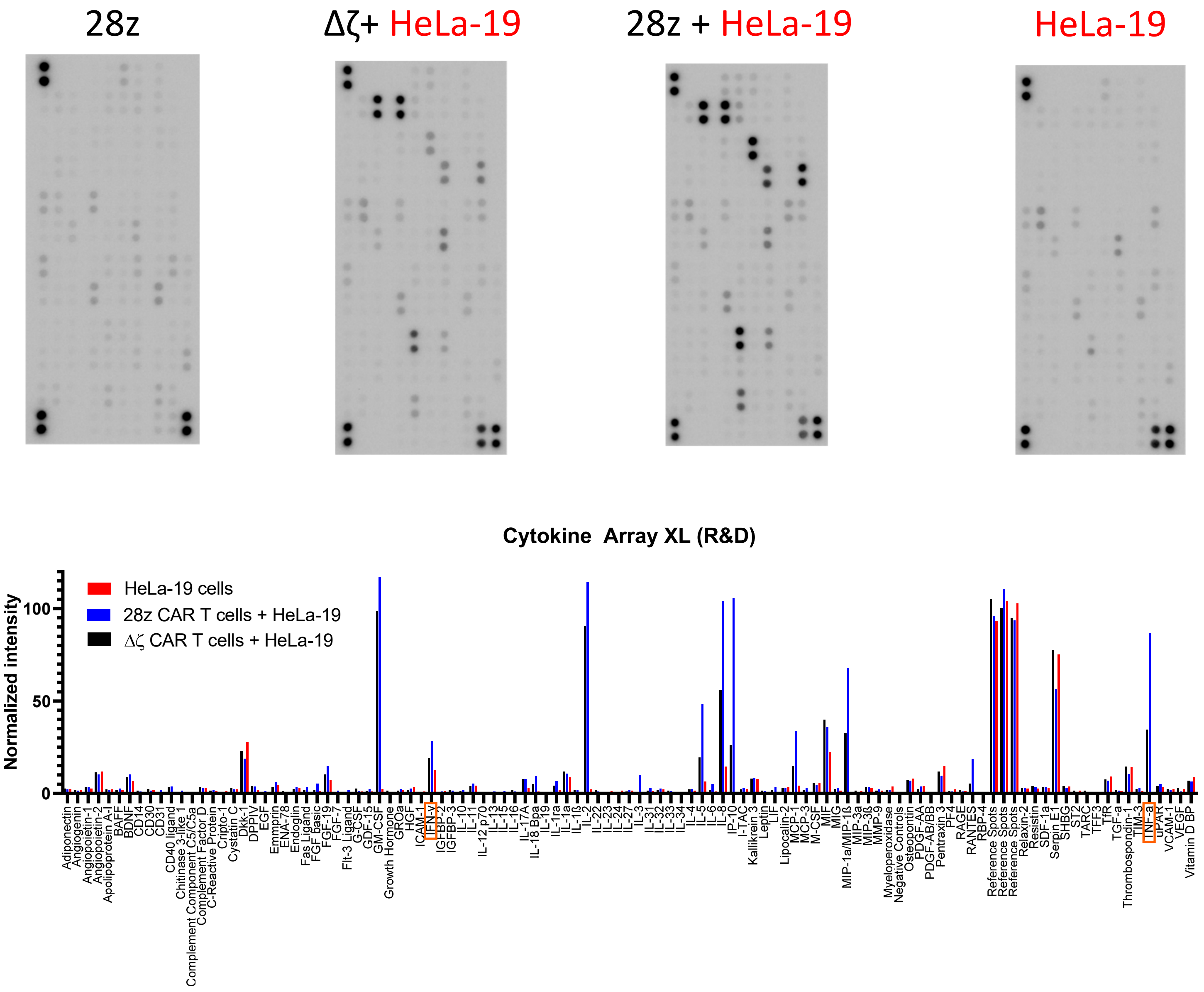

Figure S7.
Cytokine Array XL - Raw data (top) and graphed normalized intensity values (bottom) for cytokine array presented in Figure 5D and 5E. Normalized intensity values for cytokine array coordinates are presented in Table S3.
